# Deep learning based analysis of microstructured materials for thermal radiation control

**DOI:** 10.1038/s41598-022-13832-8

**Published:** 2022-06-13

**Authors:** Jonathan Sullivan, Arman Mirhashemi, Jaeho Lee

**Affiliations:** 1 Department of Mechanical and Aerospace Engineering, University of California, Irvine, USA; 2 NASA Glenn Research Center, Cleveland, OH USA

**Keywords:** Optics and photonics, Optical materials and structures, Metamaterials, Mechanical engineering, Nanophotonics and plasmonics

## Abstract

Microstructured materials that can selectively control the optical properties are crucial for the development of thermal management systems in aerospace and space applications. However, due to the vast design space available for microstructures with varying material, wavelength, and temperature conditions relevant to thermal radiation, the microstructure design optimization becomes a very time-intensive process and with results for specific and limited conditions. Here, we develop a deep neural network to emulate the outputs of finite-difference time-domain simulations (FDTD). The network we show is the foundation of a machine learning based approach to microstructure design optimization for thermal radiation control. Our neural network differentiates materials using discrete inputs derived from the materials’ complex refractive index, enabling the model to build relationships between the microtexture’s geometry, wavelength, and material. Thus, material selection does not constrain our network and it is capable of accurately extrapolating optical properties for microstructures of materials not included in the training process. Our surrogate deep neural network can synthetically simulate over 1,000,000 distinct combinations of geometry, wavelength, temperature, and material in less than a minute, representing a speed increase of over 8 orders of magnitude compared to typical FDTD simulations. This speed enables us to perform sweeping thermal-optical optimizations rapidly to design advanced passive cooling or heating systems. The deep learning-based approach enables complex thermal and optical studies that would be impossible with conventional simulations and our network design can be used to effectively replace optical simulations for other microstructures.

## Introduction

The ability to engineer how materials interact with light is at the core of the development of materials that are engineered to manage surface temperature via thermal radiation. Materials that can selectively emit or absorb thermal radiation can be engineered to passively cool beneath ambient temperatures^1,2^ or to heat radiatively^3,4^. Radiative heating and cooling depend on two spectral regions: visible (VIS) to near-infrared (NIR) and the mid-infrared (MIR) respectively^1^. Thermal absorption for a surface exposed to the sun is defined by the solar/NIR spectrum from λ = 300–2500 nm, whereas thermal emission depends on the temperature of the body^5^. A wide variety of topologies have been utilized for maximizing thermal absorption such as nano-domes^6^, corrugated surfaces^4^, core–shell structures^7^, and gratings^8^. Similarly, “passive-cooling structures”—surfaces that have significant thermal emission with limited solar absorption and can cool beneath ambient temperatures^9^—can be engineered from materials such as polymers^2,10–12^ or corrugated graphene^13,14^. Unlike many of the solutions to radiative heating and cooling, microscale pyramid-like (“micropyramid”) surface texturing surfaces can be used to engineer either radiative cooling or heating materials^15^. Periodic micropryamid texturing on a surface induces anti-reflective properties as a result of significant light confinement by the geometry^16,17^, and has been demonstrated to enhance absorption in silicon^18–26^, nickel^3,27^, tungsten^28^ as well as for dielectrics^29^ and polymers^12^.

The design and optimization of textures to control light—such as micropyramids—can be a challenge as simulating across the available design space is a computationally demanding process that often requires dedicated numerical simulation software^30^. To compound this problem, the vast array of available materials means that for a given set of application requirements and constraints there can be a different material that is best suited to fulfill those requirements. A powerful approach that has emerged in the field of nanophotonics is the use of Deep Learning (DL) and Deep-Neural Networks (DNNs) to fill the design space and to circumvent the necessity of large time investments in simulations. Inspired by the biology and architecture of the human brain, the DL methodology is capable of high levels of non-linear abstraction from datasets^31^. DL and Machine Learning (ML) have been used, in a broad setting, to solve complex problems ranging from machine vision for self-driving vehicles^32^ to automatic speech recognition^33^ and spacecraft system optimization^34–37^. In the field of optics, DL has been used recently to predict and model plasmonic behavior^31,38–42^, grating structures^43,44^, ceramic metasurfaces^45,46^, chiral materials^47,48^, particles and nanosturctures^49–51^, and to do inverse design^31,41,50–54^. Deep-Learning has also been used extensively in the field of heat transfer for applications such as predicting thermal conductivity^55,56^ and thermal boundary resistance^57^, studying transport phenomena^58^, optimizing integrated circuits^59^, modelling boiling heat transfer^60^, predicting thermal-optical properties^44,61,62^, and addressing thermal radiation problems^63–66^.

Spectrally selective surface designs are heavily dependent upon material selection. The interaction of light with a surface is a process regulated by the complex refractive index of the material(s) involved^67,68^, and material selection is fundamental to a microstructure’s performance. Different material classes such as metals, ceramics, polymers, and dielectrics interact differently with light, and the influence of geometry and microstructure can vary significantly even for small changes in the constituent material’s complex refractive index. A polymer, for example, has a strong mid-infrared response as a function of its complex refractive index, but due to the extinction coefficient of ~ 0 in the VIS–NIR, it is optically transparent. To manipulate the optical properties in those wavelengths, another material needs to be included in the polymer matrix^10,69^. To provide a comprehensive thermal optimization, we need to be able to exhaustively search across a material catalog to find what material and geometry combination are best suited for the thermal design requirements.

In this paper we propose a methodology based on a DNN to predict the optical properties of micropyramids across a wide design space of geometries, wavelengths, and, most importantly, materials. As opposed to many other studies that provide a deep learning approach to a structure with a single material^38,52^, a geometry with fixed materials^44,47,51^, or a material input defined by one-hot encoding with a random forest^50^, our DNN is designed to predict the optical properties of a vast array of materials and is not constrained by material input. While there are many available machine learning methods^42,50,61,70–74^, we choose to utilize the deep neural network approach due to the method’s input flexibility, scalability, and the ability to extrapolate outputs from unseen inputs.

The model we present can predict the transmissivity, reflectivity, and emissivity of micropyramids across a diverse library of materials. = Our model emulates finite-difference time-domain (FDTD) simulation outputs by predicting spectral properties for a combination of the plane-wave source wavelength, geometric properties of the texture, and material. The model differentiates materials by taking discrete material inputs derived from the complex refractive index and subsequently builds relationships between the material inputs and the geometry and wavelength to predict the transmissivity and reflectivity. From the predicted optical data, we make thermal predictions for the texture’s thermal emission and absorption performance. For a given material, there is a vast optical property design space afforded by a microstructure. By using the network to search across a library of materials, we can identify material and geometry combinations that can best optimize a set of thermal conditions. We can rapidly perform exhaustive searches across a material database and geometric design space to find optimum combinations, a process that would be too computationally expensive with previous micropyramid optimization approaches. While we apply our methodology to micropyramid structures, our methodology has wide applicability for neural network designs that can replicate and effectively replace optical simulations for metasurface and microstructured surface optimization.

## Results

### Model training and design

Solutions using the FDTD method, while accurate, are time consuming. Optimizing the spectral properties of a microstructure can be a challenge due to the number of simulations required. We employ a deep neural network architecture that can estimate the simulation outcome to predict optical properties rapidly and accurately. We design a network that can predict across the geometric design space of a micropyramid for a given minimum and maximum wavelength (λ_min_ and λ_max_) and is capable of modeling and predicting the behavior of micropyramids constructed of an array of materials. Once the model is trained, the prediction phase is nearly instantaneous. Thus, if the model’s predictions are accurate, we can perform accurate optimizations in the span of seconds and mitigate the necessity of additional computationally expensive simulations.

**Figure 1:**
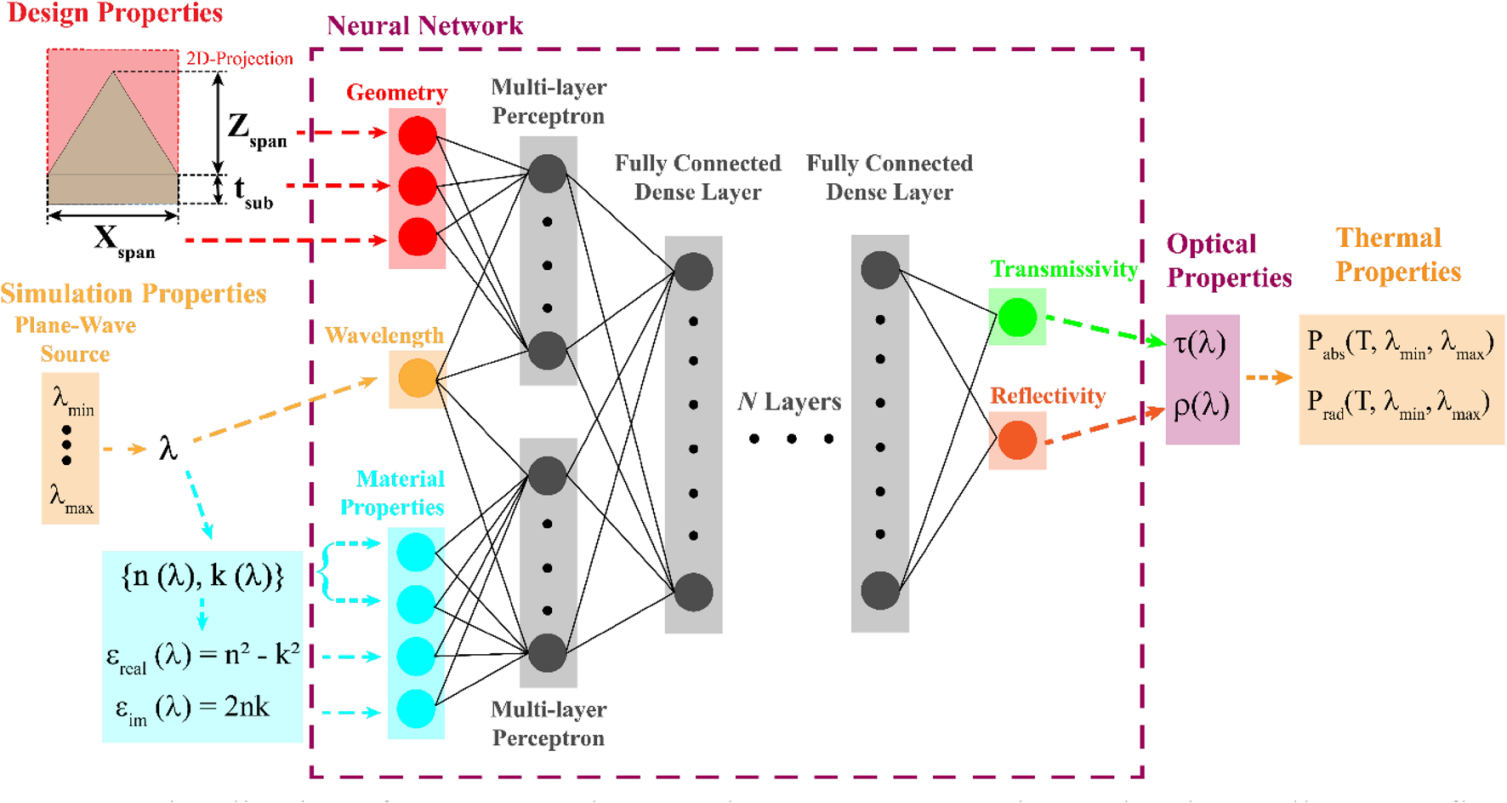
Visualization of Deep-Neural Network (DNN) construction and and overall process flow.

Our model is trained, validated, and tested on a dataset constructed of data compiled from 35,500 different simulations from Lumerical’s commercially available 2D/3D FDTD solver. The simulation framework provides exact solutions for Maxwell’s equations across a finite element mesh and we can extract the dispersion and absorption from the results^75,76^. For this work, all simulations are calculated in 2D to minimize simulation time and generate large datasets for each material. 2D FDTD simulations deliver accurate results for micropyramid geometries, but slightly overestimate the emissivity when compared to a more realistic 3D simulation^15^. We chose to simulate our periodic micropyramid structures using FDTD instead of a semi-analytical approach such as RCWA^77,78^ due the accuracy of the FDTD method as well as the scalability and applicability of FDTD beyond the geometrically simple structures shown in this work. The simulations are based upon the geometry shown in Fig. 1, with key independent geometric parameters: the triangle base span (x_span_), height (z_span_), and substrate thickness (t_sub_). We utilize periodic boundary conditions for our simulations: the structure shown in Fig. 1 occupies the entire unit cell. Additionally, as micropyramids have been shown to demonstrate omnidirectional optical properties^3,15,18,26^, we do not vary the source angle of incidence or polarization. For this work we assume that Kirchhoff’s law is valid and the emissivity we calculate from the simulations is derived from α = ε = 1 – R – T, where reflectivity (R) and transmissivity (T) are calculated from power monitors above and below and domain respectively and where absorptivity (α) is synonymous with emissivity (ε). For each material, we generate a uniformly distributed random matrix of x_span_, z_span_, and t_sub_ variables and run the simulation with a plane-wave injection source that ranges from λ_min_ to λ_max_. Details on the dataset distributions can be found in the supplementary materials. The size of the randomly generated geometric property matrix corresponds to the number of simulations, with the randomness generally ensuring that simulations in a dataset have a unique combination of the three geometric variables. While the x_span_ and z_span_ coordinates are randomly generated with values ranging from 0 to 10 um, the range of t_sub_ and λ_min_ to λ_max_ properties are selected based on the material. A detailed description of the simulation domain and setup are in the Methods section. Additionally, our simulations assume that there is no surface roughness or additional hierarchy to maintain simulation simplicity.

The geometric input parameters used in simulations and input into the DNN are the independent parameters x_span_, z_span_, t_sub_. We assume periodic boundary conditions for a unit cell that contains a single micropyramid with the specified geometric parameters. We divide the wavelength spectrum used in simulation into a set of single inputs. Each wavelength point has a set of λ-dependent n, k, ε_real_, and ε_im_. The material properties are used as inputs to one multi-layer perceptron (MLP) and the geometric properties/wavelength are grouped as inputs for another MLP. The MLPs concatenate and connect to a larger DNN structure. The outputs of the DNN are a reflectivity and transmissivity point corresponding to λ.

The architecture of the neural network shown in Fig. 1 is designed to emulate the critical simulation inputs that influence the computed optical properties. In total, our network employs 8 inputs: x_span_, z_span_, t_sub,,_ λ, n, k, ε_real_, ε_im._ These inputs follow three classifications: geometric parameters, wavelength, and material data. The geometric parameters are x_span_, z_span_, and thickness of the substrate under the surface texture (t_sub_). We include the substrate thickness to capture the optical property behavior with respect to the thickness so that our model can more accurately interpret and predict the behavior of transmissive materials. The second input classification is the injection wavelength (λ). The wavelength is the fundamental determining factor that links the output and material data together. In a FDTD simulation, each frequency/wavelength point we solve at has a corresponding set of optical properties (ε, R, T) so to emulate that behavior we utilize a single wavelength point as a network input. The solution to Maxwell’s equations is not sequentially dependent, meaning that we can separate a large, simulated wavelength spectrum into smaller groupings of inputs for the neural network. Previous network designs employed the full simulation wavelength spectra and the corresponding wavelength dependent material properties, but we found that dividing the full-spectrum simulations into single wavelength inputs yields much more accurate results. Details on our design iteration can be found in the supplementary materials.

Corresondingly, the FDTD method uses the complex refractive index to differentiate between materials. At each wavelength point of the solution, there is a matching refractive index value (n) and extinction coefficient (k). The third grouping of the neural network’s input parameters—the material properties—enable the DNN to differentiate materials similar way to how a FDTD simulation would. To better strengthen the connection between material properties and the output, we include two correlated parameters—the real (ε_real_) and imaginary permittivity (ε_im_), shown in Fig. 1. Compared to using only normalized n and k inputs to differentiate materials, the inclusion of the correlated parameters strengthens the connections between the material input and the output optical properties, enabling higher prediction accuracy for materials not included in the training of the model. The output of the neural network is the reflectivity and transmissivity that correspond to the wavelength input and material/geometric properties. This design emulates the output of the power monitors used in the FDTD simulations. Predicting all three optical properties is unnecessary as—assuming Kirchhoff’s law is applicable—we calculate the emissivity from the other two properties. To further enhance the connection between input and output, we utilize two smaller multi-layer perceptron (MLP) architectures that allow the model to build connections with the geometry/wavelength and wavelength/material properties respectively. The outputs from these MLPs are fed into the larger DNN structure. The uncoupled MLP structures are implemented to increase the connections between the inputs and to develop separate non-linear relationships between the key independent parameter (λ) and the geometric information and the material information. The concatenated output of the MLPs is fed as an input to the larger and fully connected sequential DNN structure. In our design process, we have found that this methodology has led to increased accuracy in extrapolating optical properties for new materials.

**Figure 2:**
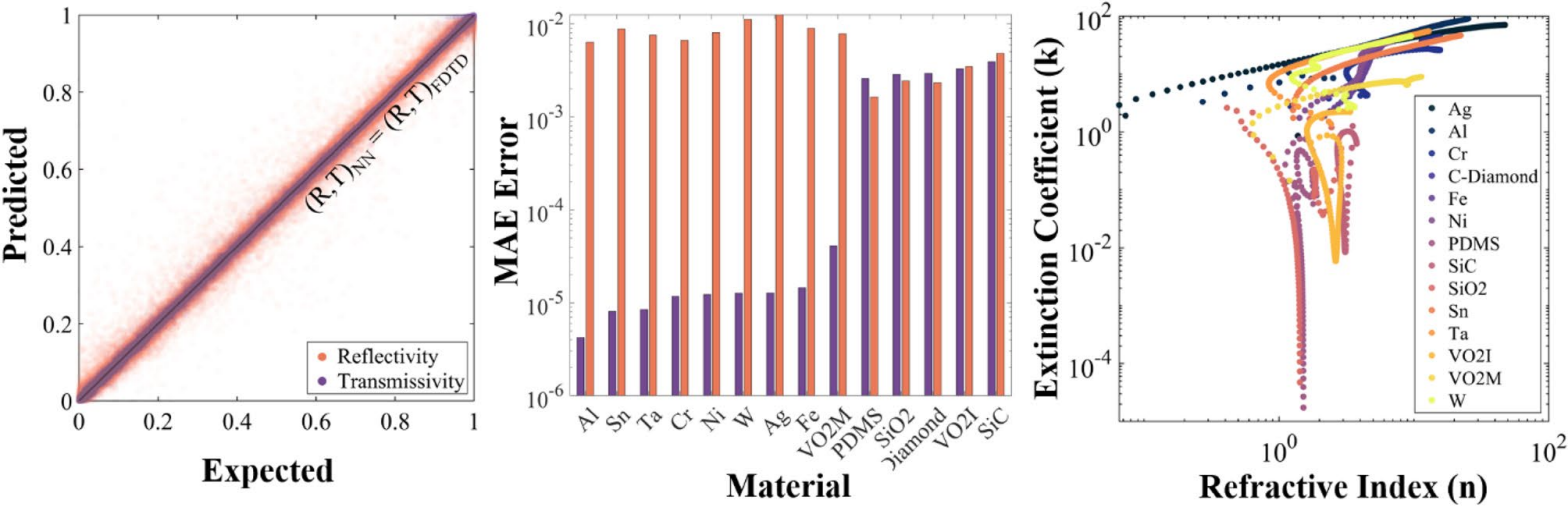
(**a**) neural network predictions of the optical properties compared to the properties obtained from FDTD simulations plotted for the test dataset. (**b**) By material average mean absolute error (MAE) for reflectivity (orange) and transmissivity (blue) separately for the test dataset. No error exceeds 0.01, the overall test dataset has an MAE of 0.0034. (**c**) The extinction coefficient (k) vs the refractive index (n) for all of the materials included in the test dataset, highlighting the differences between the materials used in the training of the network.

The DNN is trained using the FDTD generated datasets and allow it to learn and predict the non-linear relationships between the input geometry, wavelength, material properties, and the output spectra. The simulation data is divided into three separate subgroups: training, validation, and testing, which carry a 70/20/10 split respectively. We use the training and validation data in the model generation process. The test dataset—unseen during training—is used to evaluate the performance and accuracy of the network in interpolating optical properties for new geometric and wavelength combinations. The training/test datasets encompass simulations from 14 different materials of widely varying complex refractive index, including metals (Ni/Ag/Al/Cr/Fe/Sn)^79–81^, refractory metals (Ta/W)^79,82^, a phase-change material (VO2 Metallic/Insulating)^83^, a polymer (PDMS)^84^, a semiconductor (SiC)^85^, a ceramic (SiO2)^79^, and a material with a near zero extinction coefficient across a wide spectrum (Diamond)^86^. The network predictions vs the simulation results for the test dataset are shown in Fig. 2a,b. The diverse set of materials enables the network to interpret a wide range of n and k inputs—including extreme values—during the training process. The values of the complex refractive index are plotted in Fig. 2c, to highlight the differences between the materials in the training/validation/test datasets.

The test dataset does not contain new material data, but it includes geometric combinations that the model has not seen in training. Our model demonstrates an ability to predict with an extreme accuracy new geometric/wavelength combinations made of materials included in the training process. The error between the prediction and simulation values is plotted in Fig. 2a and broken down by material in Fig. 2b. The mean absolute error (MAE) for the training and validation sets are 0.0034 and 0.0035 respectively. These error values correspond to an MSE error for the training/validation datasets of 1.22e−4 and 1.34e−4 respectively. The test dataset has an MAE and MSE error of 0.0034 and 1.53e−4 respectively. While some outlier predictions do exist, as visualized in Fig. 2a, the error by material in Fig. 2b validates that our model is interpolating the optical properties for “seen” materials with high efficacy. An apparent relationship from Fig. 2b is that transmissive materials show a larger error in the predicted transmission, and metallic materials show an increased error in reflection. This is a manifestation of the role of the extinction coefficient, with a high extinction coefficient leading to reflection dominated optical properties and low extinction coefficient leading to transmission dominated optical properties. For some materials with a low extinction coefficient (k <  < 1), geometry has little to no influence on the reflection and t_sub_ is the only geometric parameter that determines the optical properties. This relationship necessitates that the design of the network correctly connects the material properties, wavelength, and geometry, to make accurate predictions for any arbitrary material not included in training.

The small differences in error between the test/evaluation datasets and the training/validation training errors verify our network can predict the optical properties for inputs within the design limits with a high degree of accuracy. Additionally, the minimal difference in error between the test and training/validation datasets allows us to conclude with a high degree of certainty that our model is not overfitting during training. We validate this assumption by examining the overlap in geometric parameters between the test and training/validation datasets, shown in the supplementary materials. We discuss the precise architecture, details on the hyperparameter optimization, network parameters, network architecture optimization process, etc., of the model used to achieve these results in the Methods section.

### Optical predictions for select materials unseen in training

The network’s input design—with distinct material inputs, wavelength, and geometric parameters—enable our network to dynamically predict the optical spectra of micropyramids made of materials that are not included in the training process. We first test our network’s capacity to predict the optical properties of new materials with two new datasets: a metal (Titanium)^79^ and a ceramic (Alumina, Al_2_O_3_)^87^ dataset comprised of 1500 simulations each. These materials are not used in the training or validation process, and they were explicitly chosen as Ti/Al_2_O_3_’s complex refractive index values significantly differ from the materials used in training. Comparisons of the refractive indices used in training to those predict the titanium and alumina datasets are shown in the supplementary materials. These datasets were generated with the same methodology as before and each simulation has a unique combination of t_sub_, x_span_, and z_span_. After making predictions with a trained neural network that does not include any Titanium or Alumina data in training, we generate a different model that includes 10 randomly selected simulations from the alumina and titanium datasets (< 1% of the simulations) to compare the prediction accuracy when a small amount of data is included in the training process.

**Figure 3:**
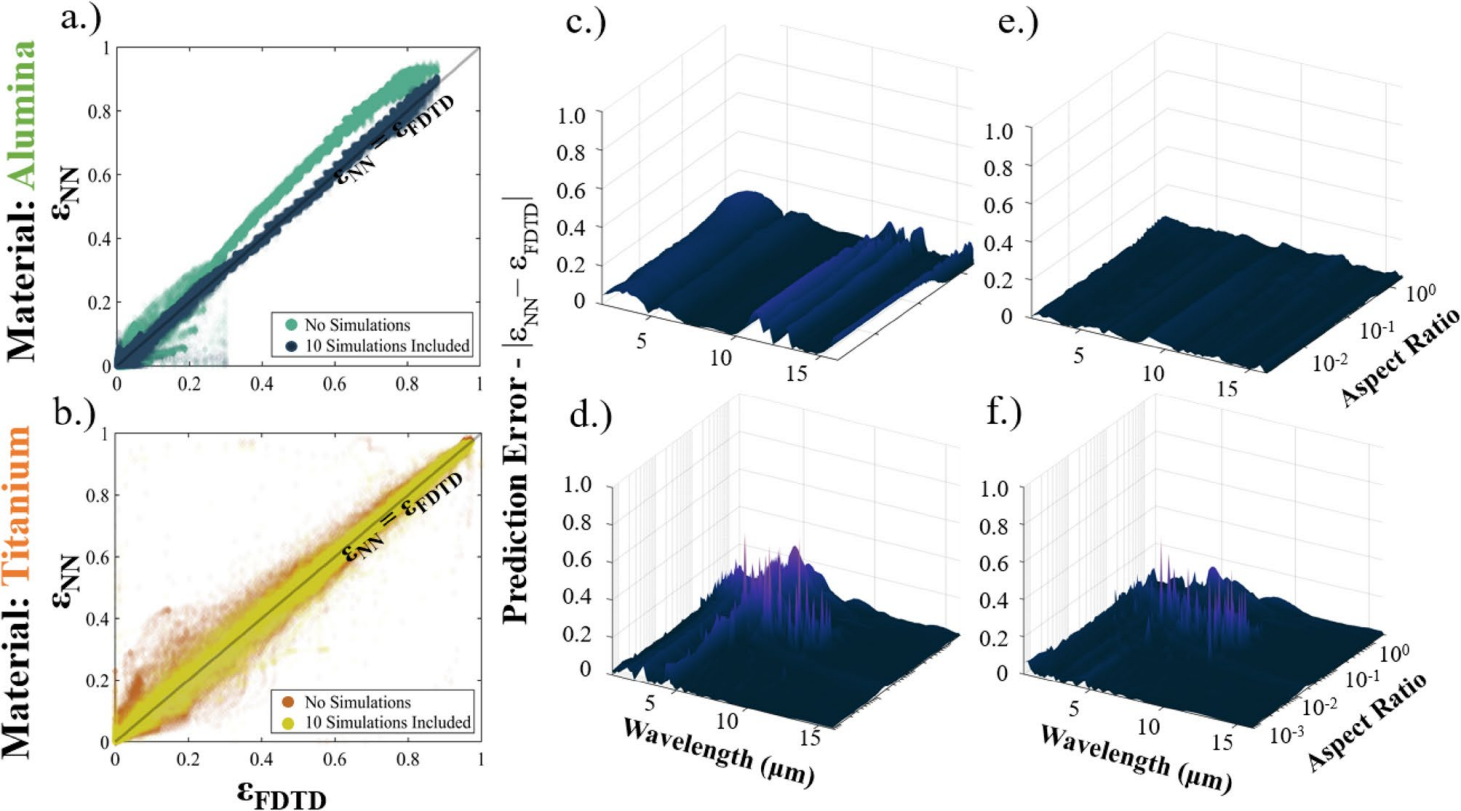
Neural-network predictions for two materials (Ti/Al2O3) that are not used in the in the training process. (**a**, **b**) The predicted optical properties vs. the FDTD computed properties, with and without 10 simulations included in training for alumina and titanium. Surface plot of the absolute error between prediction and simulation with no simulations included (**c**, **d**) and with simulations included in training. The wavelength is on the x-axis and the geometric information is visualized with the aspect ratio on the y-axis. Including 10 simulations (1% of the dataset) dramatically reduces the error in the alumina to a near zero value across all wavelengths and aspect ratios. For Ti, the resonance driven peaks in the low-aspect ratio structures are reduced and the error in all other sections becomes approximately zero.

Figure 3a,b plots the predicted optical properties by FDTD simulation vs the neural network predictions. The MAE between prediction and simulation for the alumina and titanium datasets are 0.0175 and 0.0131 respectively. Broken down by individual output, the MAE_Reflectivity_ is (0.026, 0.0063) and MAE_Tranmissivity_ is (6.01e−5, 0.028) for titanium and alumina respectively. The error in the reflectivity and transmission mirrors the results in Fig. 2b – metallic materials have reflection driven optical properties with geometry and show a very low error in transmission. Conversely, the relationship between the substrate thickness and extinction coefficient of alumina leads to non-zero transmission, with geometry playing a reduced role in determining the reflection and transmission properties. In Fig. 3c,d we compare the absolute difference between the neural network and FDTD predicted emissivity for the alumina and titanium datasets. Across both the geometric and wavelength space, we observe a high degree of accuracy in the neural network predictions. The exception to this is a significant deviation in the titanium dataset (Fig. 3d) that occurs in a region with high material/geometry specific resonance. Similarly, the network minorly underpredicts the role of transmission in Al_2_O_3_, leading to the observed prediction differences. Despite these differences, the model is clearly able to differentiate material in a meaningful way and extrapolate accurately beyond the dataset used in training.

To improve the prediction accuracy, we examine what occurs when we include a seemingly trivial amount of simulation data from the “unseen” materials in the training process. We select 10 simulations at random from the Ti and Al2O3 datasets (< 1%) and include them in the training/validation/test datasets. Figure 3a,b and Fig. 3e,f underscore that the small inclusion of data has a large impact on the prediction accuracy. The overall MAE score becomes (0.0073, 0.0049) while MAE_Reflectivity_ improves to (0.014, 0.004) and MAE_Tranmissivity_ improves to (7.69e−5, 0.0058) for titanium and alumina when 10 simulations of each are included in the training dataset. These error values are close to those shown in Fig. 2b for the materials in the test dataset, indicating that only a small amount of simulation data is required to calibrate the model for a new material. Figure 3e,f demonstrates that even this small amount of data—while not enough to completely remove error—effectively reduces prediction error throughout, even in the highly erroneous resonant region of Ti. While the prediction error from completely unseen data is excellent, including a small number of simulations align the accuracy of the “unseen” materials with the accuracy of the much larger datasets included in training.

### Optical predictions for a library of materials unseen in training

To further demonstrate the capability of our model to provide accurate optical predictions for microstructures made of materials outside of the scope of the model’s training, we compare the network’s predictions to simulation results for 23 additional materials that were not seen in the training process. As many of these materials require much more time to simulate each geometric combination, we only perform 100 simulations for each material, for a total of 2300 additional simulations. The materials included in this library range dramatically in material properties, with the complete material list and compilation of prediction accuracy shown in Table 1.

**Table 1:** MAE Errors in the reflection and transmission without no simulations included in the training data and 5 simulations included in training.

Material	MAE reflection		MAE transmission		Error difference	
	No simulations	5 Simulations	No simulations	5 Simulations	ΔR	ΔT
Au^88^	0.0637	0.0456	2.63E−05	3.88E−05	− 0.018	1.25E−05
B_4_C^89^	0.0394	0.0098	0.0542	0.0091	− 0.030	− 0.045
BaF_2_^90^	0.0040	0.0009	0.0907	0.0053	− 0.003	− 0.085
Be^91^	0.0086	0.0067	5.01E−06	3.72E−06	− 0.002	− 1.29E−06
C^92^	0.0972	0.0124	3.11E−04	3.12E−05	− 0.085	− 2.80E−04
Cu^79^	0.0709	0.0880	1.07E−02	1.80E−05	0.017	− 0.011
GaAs^79^	0.0249	0.0106	0.0927	0.0186	− 0.014	− 0.074
Ge^79^	0.0869	0.0216	0.0964	0.0094	− 0.065	− 0.087
In^93^	0.0312	0.0223	6.95E−06	4.30E−06	− 0.009	− 2.65E−06
InAs^94^	0.0036	0.0030	0.0849	0.0121	− 0.001	− 0.073
InP^94^	0.0098	0.0053	0.0722	0.0126	− 0.005	− 0.060
Li^95^	0.0570	0.0297	8.23E−04	8.21E−04	− 0.027	− 2.04E−06
Mg^96^	0.0434	0.0295	5.84E−06	4.14E−06	− 0.014	− 1.69E−06
Mo^82^	0.0316	0.0296	7.39E−06	5.04E−06	− 0.002	− 2.35E−06
Nb^97^	0.0156	0.0154	4.55E−06	3.13E−06	0.000	− 1.42E−06
Os^98^	0.0282	0.0291	1.21E−04	1.20E−04	0.001	− 1.70E−06
Pd^91^	0.0168	0.0109	1.48E−05	1.02E−05	− 0.006	− 4.56E−06
Pt^79^	0.0096	0.0086	5.49E−06	4.32E−06	− 0.001	− 1.17E−06
Rh^79^	0.0106	0.0125	8.07E−06	6.89E−06	0.002	− 1.18E−06
Si_3_N_4_^87^	0.0021	0.0021	0.0321	0.0046	0.000	− 0.028
TiO_2_	0.0006	0.0016	0.2003	0.0359	0.001	− 0.164
Zn^90^	0.0554	0.0203	1.77E−05	1.34E−05	− 0.035	− 4.31E−06
Zr^90^	0.0141	0.0122	3.21E−05	2.17E−05	− 0.002	− 1.04E−05

The relative error for each material is shown, with nearly all materials showing a significant decrease in error as a result of the small amount of data being included.

**Figure 4:**
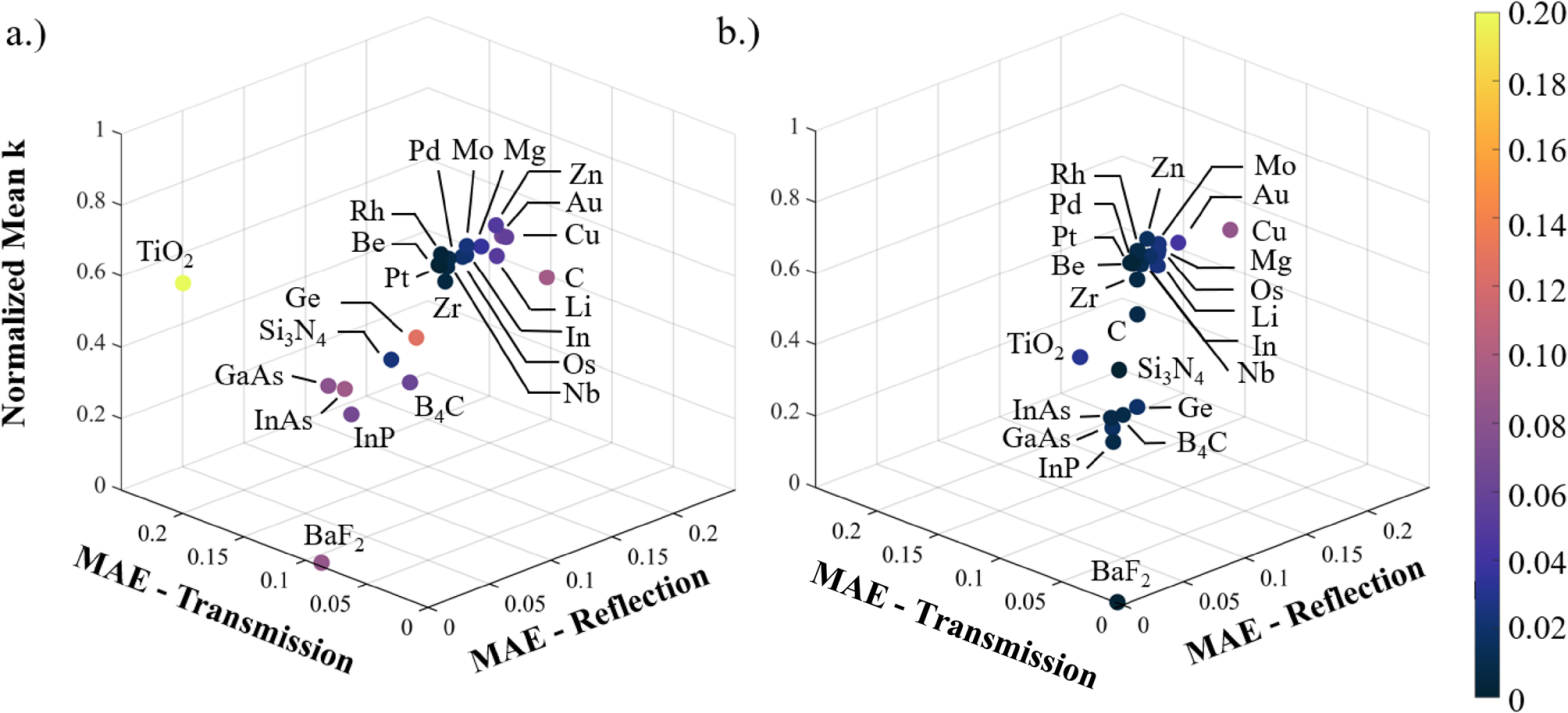
MAE for the transmission and reflection predictions compared to FDTD simulations for the 23 unseen library materials. (**a**) Plotted error when the materials are completely “unseen” and (**b**) after 5 simulations for each material are included in the training/testing/validation process. The log and then linearly normalized average extinction coefficient is shown in the z-axis, pointing to the role of the material in predicting where the error will occur. The error’s (x,y) distance from an MAE error of zero is shown with the color bar. Including 5 simulations systematically reduces the prediction error for the rest of the dataset, indicating that very little data is needed to calibrate the model for new materials and lead to accurate predictions.

The error between the predicted optical properties and simulated optical properties for the unseen material library is plotted in Fig. 4a, with the errors shown in more detail in Table 1. The only material with an MAE > 0.1 is TiO2, with a transmission prediction error of 0.2003. Low extinction coefficient materials generally exhibit more error in transmission and high extinction coefficient materials exhibit a larger error in reflection. The results indicate that while the neural network does not perfectly replicate the physics of the FDTD simulations, it is nevertheless accurate in making predictions for materials that vary significantly from those used in training– the overall mean average error across all 23 materials is 0.0279.

We can improve this accuracy and calibrate the model by including a small number of simulations in the training/validation/test datasets. Here, we choose 5 random simulations from each of the 100 to include in the training/validation/test datasets. After training the model on this data, we show the improvement to the predictions in Fig. 4b. Despite using only 5% of the simulations contained in these materials’ datasets, the small calibration data has removed much of the error on every material. The combined MAE score after 5 simulations are included in the training process is 0.0118. The increase in accuracy provides further validation that our model has connected the inputs to the outputs via the simulation physics well enough that it only requires a small amount of calibration data to produce extremely accurate results across the rest of a material’s latent design space.

### Material selection algorithm and thermal optimization

We apply the strength of our network architecture by using it to make optical predictions for a library of materials. We use the resulting optical predictions from the neural network to perform thermal optimization and search for the material and geometry that best optimize our selected thermal conditions. In total, we pass along 41 materials into the trained neural network. The materials cross a wide spectrum and include all the materials that were included in training, titanium/alumina, and the 23 other materials that are unseen by the network during training.

To fully demonstrate the speed of our network and how comprehensive we can be in searching the latent design space, we generate a grid of coordinates (xspan, zspan) that span from 0 to 10 um across both axes in increments of 0.1 um, for a total of 10,000 geometric coordinate pairs for each material. Over all 41 materials, this correlates to a total input of outputs of 410,000 optical simulations. For each of these simulations, there are 100 wavelength points, for a total of 41 million sets of inputs to the network. The network requires approximately 25 – 40 s to predict the optical properties across all 1,000,000 synthetic simulation input sets for each material. In total, the network requires 15 to 20 min to make predictions for all 41 materials. Each individual DNN approximated simulation requires anywhere from 30 to 40 ms on our computer. The output encompasses a total of 82 million datapoints for all 41 materials. The remarkable speed of prediction punctuates our desire to use a neural network to mostly supplant FDTD simulations, as the trained network can comprehensively predict a library of materials’ optical spectrums in minutes.

**Figure 5:**
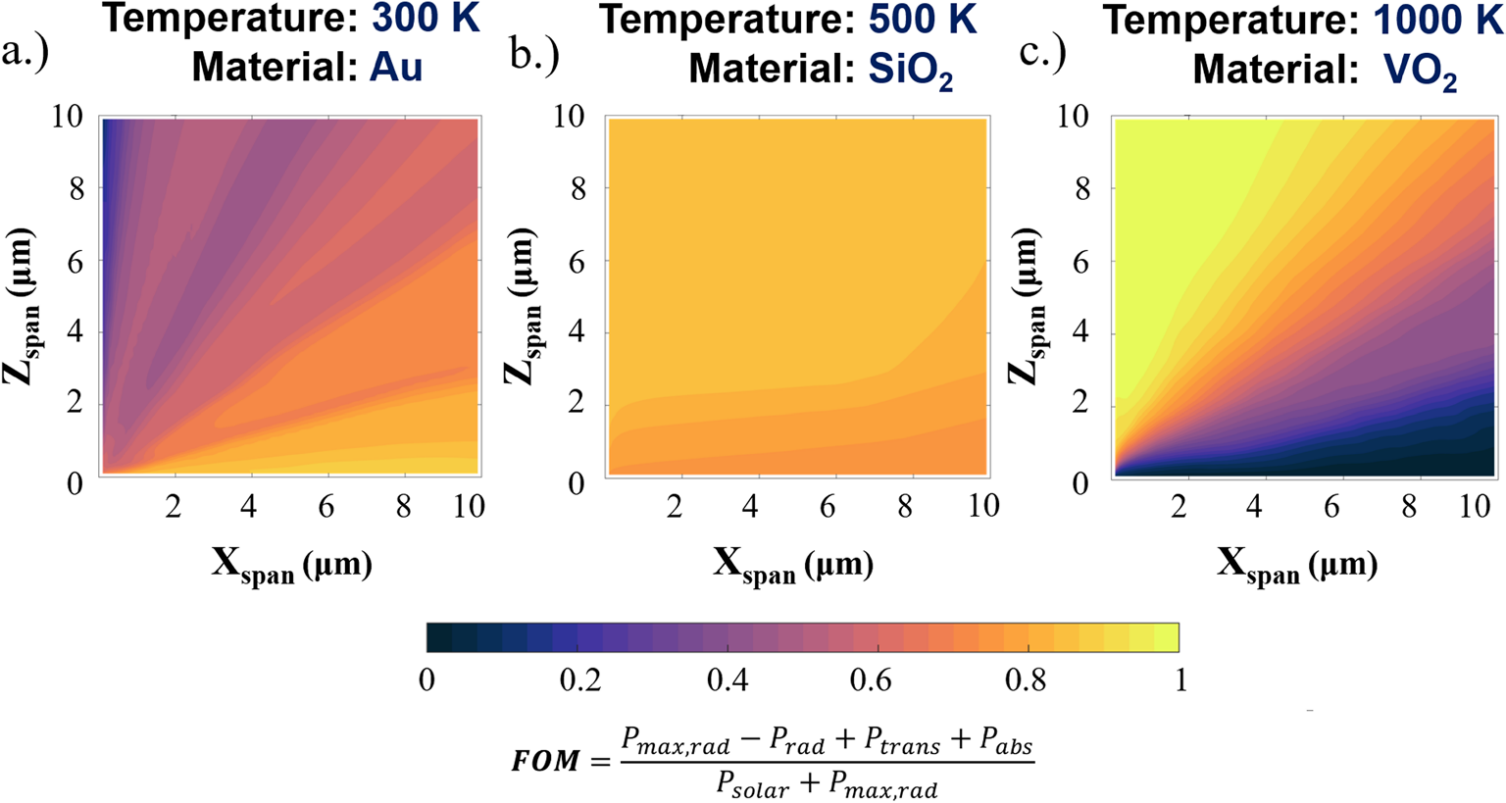
Material search algorithm identifying the most optimal microstructures for cooling at surface temperatures of 300, 500, and 1000 K based on the figure of merit defined by Eqs. (5–6). Due to the role of transmission in Eq. (5), the most optimum microstructure for cooling at room temperature is Au (FOM = 0.772) as typical cooling materials such as PDMS and SiO_2_ transmit thermal radiation in the visible wavelengths, negating cooling for a surface below. At 500 K, we identify SiO2 micropyramids as being most optimal (FOM = 0.852). At 1000 K, the algorithm identifies VO_2_ as best performing micropyramid structure (FOM = 0.982) among all 41 materials that were predicted by the network. All materials are predicted assuming a minimum wavelength of 0.3 and maximum wavelength of 16 um to capture both thermal emission and solar absorption optical properties.

We then use the DNN spectral predictions to perform a material search process to identify what materials and geometries best optimize a set of imposed thermal optimization equations. The selection of the thermal optimization equation is application specific. For sake of demonstration, the optimization we present is for high-temperature cooling. Additional optimizations using different optimization conditions are presented in the supplementary materials. The equations and thermal optimization are discussed in the Methods section. We process the thermal optimization equations for each wavelength dependent spectral property matrix to generate a figure of merit (FOM), a task that requires a significantly larger amount of computational time than the neural network’s optical predictions. Figure 5 shows the materials and Table 2 shows the geometries identified by the search process that best optimizes the cooling thermal balance defined by Eqs. (2–3) at three different surface temperatures: 300, 500, and 1000 K. The definition of Eq. (3) leads to Au being the most optimal material for a single material cooling microstructure at 300 K, a result that hinges upon the role of transmission in the equation. Despite being a transmissive material, SiO2 is identified by the search algorithm as the most optimal material at 500 K. This as a result of a balance of the transmission of solar radiation with the large thermal emission at that temperature. It should be noted that we did not change the material data inputs into the network to account for temperature variation. The design of the network material inputs, allows us to adapt our material data for different temperatures if a significant difference in the material properties is expected.

**Table 2:** Identified optimal material(s) and geometries that maximize the cooling figure of merit equation Eq. (4).

Temperature	Material	FOM	X_span_ (μm)	Z_span_ (μm)
300	Au	0.772	7.141	0.304
500	SiO_2_	0.852	1.415	5.555
1000	VO_2_	0.982	0.203	9.293

## Discussion

In contrast to FDTD simulations, which can take anywhere from minutes to hours to run, each DNN prediction takes approximately 30–40 µs/input to predict the spectra of a geometry/material/wavelength combination. Comparatively, we estimate simulating the same 10,000 parametric grid of geometries would require on average 1–3 months for the solutions to compute in FDTD via our simulation computers for each material. Accordingly, the neural network approach is approximated to be 6–8 orders of magnitude faster than traditional simulation methodologies. This estimation changes based on the available computational resources, but a tremendous benefit to a neural network driven approach is that an already trained model requires a miniscule quantity of resources to operate and make predictions. Our methodology is also scalable beyond the 2D micropyramid simulations we used in this work. We utilized 2D simulations such that we could more rapidly generate large training datasets, but our approach can be easily applied to replace or significantly reduce the reliance on simulations of more complex geometries or designs of other microstructures to increase throughput by orders of magnitude.

While our approach cannot completely replace simulations, we dramatically reduce the necessity of computationally expensive optical simulations. The use of material information and wavelength enables the model to build connections between the inputs and the physics, providing accurate predictions for a wide variety of materials that are highly dissimilar from those used in training the model. The model we show can be used to make generally accurate predictions for a material, with an overall MAE of 0.0279 for the library of completely unseen materials. Despite this accuracy, we can make more confident predictions by including a small amount of calibration data from simulations to tune the model to new physics, resonant behavior, etc., that may be present in the new material as a function of material properties or geometry. Including merely 5 simulations in training (500 datapoints) reduces our error on the remainder of the dataset to 0.0118. This indicates that our model is not merely interpolating existing material/geometry results and is making reliable predictions for materials that vary dramatically from those used to train the network.

The speed of the network combined with the ability to predict materials unused in training facilitates explorations of the design space in ways that would be impossible with traditional simulations. Figure 5 shows several specific instances of material and or geometry combinations that suit several generalized thermal balance equations, but there are near limitless combinations of temperature and environmental conditions. Our methodology allows for us to define a set of thermal conditions and search hundreds of thousands of material/geometry combinations in seconds to determine which combination yields the best result.

The network can perform thermal optimization in minutes—a task that would take years to generate a similarly sized dataset to search through using FDTD. The capability to explore the comprehensive material and geometric latent space of our problem empowers us to solve complex problems both rapidly and comprehensively. An example of this is using the network to identify optimal fabrication designs within particular constraints: for example, if the aspect ratio needs to be limited, we can identify in seconds both the material and geometric combination that provide the best expected results under the constraints. We provide an example of this in the supplementary materials. The network can also be utilized to quantify expected fabrication and experimental uncertainty by exploring the effects of nanoscale changes in the geometric parameters on the optical properties.

Ultimately, a fundamental problem facing surrogate models is the diminishing returns: to provide accurate results, more data is required, to the point where the design space has been thoroughly explored to generate the neural network model. The test dataset exemplifies this. While we can still explore minutia and small variations in geometry, a large amount of computational time was invested in generating the combined training dataset, to the point where the necessity of the neural network is diminished for these materials. Where our network is different from others that merely interpolate geometric or existing results is in the prediction of materials that have drastically different relationships between the incident wavelength, material properties, and geometry. We desire a network that can accurately extrapolate optical properties from any input material, without needing distinct and/or limiting classification methods or a large amount of new data. A particular challenge in developing the model to this end was overcoming errors in the prediction of transmissive materials. Whereas the reflection is primarily a material/geometry dependent phenomenon, transmission depends on more parameters. The inclusion of separate MLP’s, the permittivity inputs, and different normalization methods were all designed to improve the prediction accuracy of the model for both reflective and transmissive materials, ultimately improving the model’s connection to the relevant physics.

The ability of the model to take different material inputs and predict outside of its original training scope—not bound to classification—unlocks many possibilities. This includes predicting optical changes at different temperatures, enabling much more complex temperature dependent optimizations. While we do not demonstrate a reversible network in this work, the network shown could also serve as a basis for a reverse-network structure. Multiple problems—such as multiple material solutions for the same desired optical output—will need to be overcome to implement a successful reverse network capable of predicting across a wide array of materials. These insights will inform the next generation of models that move to more complex microstructures with more material, geometric, and thermal parameters.

## Conclusion

We have demonstrated a Deep-Neural Network that can emulate finite difference time domain simulation outputs that can be used for the rapid thermal and optical optimization of microstructured surfaces. The network can make accurate predictions for micropyramids across a wide array of materials and can accurately extrapolate optical properties from input data that is outside of the scope of training. Further, the network design allows us to accommodate and train on any number of materials and allows us to make predictions for the optical properties of micropyramids made of materials the model has not been trained on. We have demonstrated how our model can be used as the basis for a material search algorithm that can identify materials and geometries that best optimize a thermal environment and set of constraints. The neural network driven predictions occur at a rate 6–8 orders of magnitude faster than the simulations that were used to train the model. The network predicts the optical spectra of over 1 million simulations per minute regardless of material choice, generating output datasets in seconds that would take years to simulate in FDTD. The material search process demonstrated in this work can identify the optimal material/geometry combination across a vast latent space nearly instantaneously. Furthermore, the methodology can be easily translated to other geometries beyond micropyramids, enabling DL based models that can significantly reduce the need for computationally expensive simulations for a variety of microstructure surface textures. Our methodology effectively replaces FDTD simulations for micropyramids, decreases the time required to optimize surface conditions, and allows for more complex and comprehensive studies to explore the latent space of the problem.

## Methods

### Data and code availability

The datasets generated and/or analyzed during the current study are available in the Optical-Prediction-Neural-Network repository, [https://github.com/jmsulliv/Optical-Prediction-Neural-Network.git].

### FDTD simulations

We perform FDTD simulations in Lumerical/ANSYS’s commercially available FDTD simulation software. The unit cell shown in Fig. 1 replicates the major variables simulated—x_span_, z_span_, and t_sub_. A plane wave source with normal incidence is placed in the z-direction. For this work we do not consider angular dependence of the optical properties or of the dependence of the optical properties on the polarization angle. The injection wavelength spans a linearly spaced vector of 100 wavelength points that begins with λ_min_ and ends with λ_max_. Perfectly matched layers are applied in the direction of the injection source to prevent boundary reflection at both the top and bottom of the domain and periodic boundary conditions are placed perpendicular to the wave source. Frequency-domain field and power monitors are placed above and below the PML boundary layers to monitor reflection and transmission respectively. Emissivity is computed using Kirchhoff’s Law, α = ε = 1 – R – T. The monitors are solved at every frequency/wavelength point, leading to a one-to-one matching of the simulation output to the wave source.

For each material dataset we generate, we specify a different λ_min_ and λ_max_. The selection of these values depend on knowledge of the material data. For materials that are transmissive in UV–VIS, (PDMS/SiO2) λ_min_/λ_max_ are set to 2 um/16 um. Most metals (Ni, Al, Ag, W, Sn, Fe) are simulated with λ_min_/λ_max_ of 0.3 μm/10 μm. All other materials and some metals (VO2, Cr, Ta) are simulated with λ_min_/λ_max_ of 0.3 μm/16 μm. The unseen materials (Ti, Al2O3) have a λ_min_/λ_max_ of 0.3/16 μm and 2/16 μm respectively. Vanadium Dioxide is divided into two separate materials: that of an insulation phase (ceramic behavior) and metallic phase (metallic behavior)^83^.

The value of t_sub_ also depends on the material selection. For metals (Ni, Al, Ag, W, Sn, Fe, Ta, Cr, Ti) and SiC we simulate over a range of random t_sub_ values confined by a minimum value of 1 μm and a maximum of 3 um. For transmissive materials with a wide range of substrate dependent performance (VO_2_, SiO_2_, PDMS, Al_2_O_3_) we choose the minimum thickness to be 1 um and the maximum to be 100 μm. More information on the simulation output’s variation vs. the substrate thickness for these materials is in the supplemental section.

### Network Architecture and optimization

We use a deep neural network with fully connected dense layers as shown in Fig. 1. Our deep learning approach is built upon the open source keras library in python^99^. Our optimized DNN uses 8 fully connected dense layers with 400 neurons per layer, and both MLPs are 4 layers of 50 neurons each. Optimization of the hyperparameters is performed with the built-in hyperband optimization method^100^. We also utilize manual cross-fold validation for limited hyperparameter optimization. For training, we utilize a MSE loss function and validate/evaluate using an MAE score based on Eqs. (1, 2) respectively, where $Y_{i}$ is the predicted value.

1 $$ MSE = \frac{1}{n}\mathop \sum \limits_{i = 2}^{n} \left( {Y_{i} - \widehat{{Y_{i} }}} \right)^{2} $$

2 $$ MAE = \frac{{\mathop \sum \nolimits_{i = 1}^{n} \left| {Y_{i} - \widehat{{Y_{i} }}} \right|}}{n} $$

Adam is the optimization engine used for the network training. To minimize overfitting, we utilize L2 regularization in the training and validation process, in addition to utilizing early-stopping, checkpoint save, and reduce learning rate on plateau callbacks.

### Datasets and normalization

All datasets used by the neural network are derived from FDTD simulation inputs and outputs directly. For each material in the training/validation/test dataset, we simulate a minimum of 1,000 individual combinations of x_span_, z_span_, and s_tub_. We generate a uniformly distributed random matrix for each of the geometric properties to use as inputs for the simulation. The simulation wavelength and n and k values are taken from each simulation and split into sets of input data, spanning a total of 8 neural inputs (n and k are converted into ε_real_ and ε_im_). The simulation output is 100 emissivity and 100 reflectivity points that one-to-one match the simulation wavelength vector, which is divided into pairs for each λ. For this work we utilize several normalization methods depending on the input dataset. X, Z, and λ are considered uniform, and a simple linear normalization is applied to each separately using Eq. (1). For the refractive index (n) we use a log-linear normalization, using Eq. (4) with α = 0 and then Eq. (3) to bring the values between 0 and 1.

3 $$ x_{norm} = \frac{{x - x_{min} }}{{x_{max} - x_{min} }} $$

4 $$ x_{norm} = \log_{10} \left( {x + \alpha } \right) $$

The distribution of k, t_sub_, ε_real,_ and ε_im_ pose a more significant normalization challenge. The ε_real_ permittivity value is of particular concern due to the negative values induced by −k^2^. The dataset distribution before and after normalization for each input is shown in the supplementary materials. A fundamental problem faced is that optically, the difference between k = 1e−4 and 1e−3 is not mathematically large, but the difference does have a large impact on the transmission behavior through the substrate. Thus, the data is grouped near 0 but we need to differentiate values in a meaningful way to distinguish the physical behavior of each material. Log normalization reduces the severity of the weighted inputs but does not solve it. Thus, for these variables, we turn to more complex normalizations. For this work, we utilize quantile normalization with sklearn’s built in quantile transformer, to generate a uniform distribution of inputs for k, t_sub_, ε_real,_ and ε_im_. To ensure our values for all materials stay between 0 and 1 on all inputs, we normalize all of the simulations together. This is done to have consistent normalization, and the complete dataset (all simulations—unseen, library, and train/val/test) is included with our github before and after normalization.

We combine 35,500 FDTD simulations for micropyramids made of 14 different materials to form our training, validation, and test dataset. We follow a 70/20/10 percentage split respectively. The test dataset is used to evaluate the performance and overfitting of the model and it is not seen by the network in the training process. The prediction accuracy of the optimized network architecture is shown for all 13 materials in the test dataset in Fig. 2. We shuffle the complete dataset every time the model is run or generated such that the training, validation, and test datasets are never identical from iteration to iteration. We utilize several other datasets in the grading our of model and the prediction of performance. The prediction performance of the model for unseen materials is evaluated with normalized datasets constructed of 1500 titanium and alumina FDTD simulations, and we have 100 simulations for each material in the 23 unseen material library. In total, our combined dataset used for normalization contains 40,300 2-D FDTD simulations in 41 different materials. Our thermal predictions shown in Fig. 4 are generated from “gridpoint” neural inputs where the only variation between the inputs for each material are the x_span_, z_span_ , and t_sub_ neurons that follow a meshgrid of coordinates. We take the outputs for each synthetic combination of x_span_, z_span_, and t_sub_ and use that to predict geometric dependent thermal performance for each material’s generated input grid.

### Thermal optimization

While we can choose to define thermal optimization equation for specific applications such as radiative cooling or heating, high temperature cooling, etc., for this work we use a simple relation for easy comparison in the unseen material predictions. The cost function used in this work neglects solar absorption and focuses only on maximizing thermal emission. We define the objective function with the heat transfer balance,

5 $$ C_{cooling} = { }\frac{{P_{{max,{ }rad}} - P_{rad} + P_{abs} + P_{trans} }}{{P_{max,rad} + P_{solar} }} $$

6 $$ C_{cooling} { } = \frac{{\mathop \smallint \nolimits_{{\lambda_{min} }}^{{\lambda_{max} }} I_{BB} \left( {\lambda ,T} \right)d\lambda - \varepsilon \left( \lambda \right)(\mathop \smallint \nolimits_{{\lambda_{min} }}^{{\lambda_{max} }} I_{BB} \left( {\lambda ,T} \right)d\lambda + \mathop \smallint \nolimits_{{\lambda_{min} }}^{{\lambda_{max} }} I_{AM1.5} \left( \lambda \right)d\lambda ) + T\left( \lambda \right)\mathop \smallint \nolimits_{{\lambda_{min} }}^{{\lambda_{max} }} I_{AM1.5} \left( \lambda \right)d\lambda }}{{\mathop \smallint \nolimits_{{\lambda_{min} }}^{{\lambda_{max} }} I_{BB} \left( \lambda \right)d\lambda + \mathop \smallint \nolimits_{{\lambda_{min} }}^{{\lambda_{max} }} I_{AM1.5} \left( \lambda \right)d\lambda }} $$

where P_max,rad_ is the maximum amount of blackbody radiation that can be emitted by the surface, P_rad_ is the emitted radiation, P_abs_ is the amount of absorbed solar radiation, P_trans_ is the amount of transmitted power through the surface, and P_solar_ is the amount of power available to absorb from the sun. P_abs_ and P_trans_ cannot be larger than P_solar_, as defined by the integrals in Eq, 4. We do not include the effects of atmospheric emission in the heat balance equation to maintain a simple relationship between the maximum emission and achieved emission by the surface in the optimization process. The heat transfer equation shown in Eq. (5) is a cost-function equation that prioritizes cooling performance when subjected to solar radiation. Preferentially, the surface should reflect all incident radiation while maximizing thermal emission. As we are only considering a single material system, we include a term that accounts for transmitted power. Some materials (such as PDMS or SiO2) are good emitters but would allow solar radiation to pass through, leading to deceptive performance unless a term that accounts for transmission is included. In our search process utilizing these equations, we are attempting to minimize the cost function.

For this work, we present the results in terms of the figure of merit—as seen from the coordinate grid contour plots of Fig. 5a–c which is defined by Eq. (7) as,

7 $$ FOM = 1 - C $$

## Supplementary Information

Supplementary Information.
