# Supplementary material for: Deep learning based analysis of microstructured materials for thermal radiation control
Source: Sci Rep. 2022 Jun 13;12:9785. doi: 10.1038/s41598-022-13832-8 (PMC9192759; doi:10.1038/s41598-022-13832-8)
Supplement: Supplementary file 1 — Supplementary Information. [file 41598_2022_13832_MOESM1_ESM.docx]

**Deep Learning Based Analysis of Microstructured Materials for Thermal Radiation Control**

Authors: Jonathan Sullivan^a^, Arman Mirhashemi^b^, Jaeho Lee^a*^

*^a^Department of Mechanical and Aerospace Engineering, University of California Irvine, United States*

*^b^NASA Glenn Research Center, Cleveland OH, United States*

*Corresponding author: jaeholee@uci.edu

**Supplementary Information**

**Section S1. Material Data**


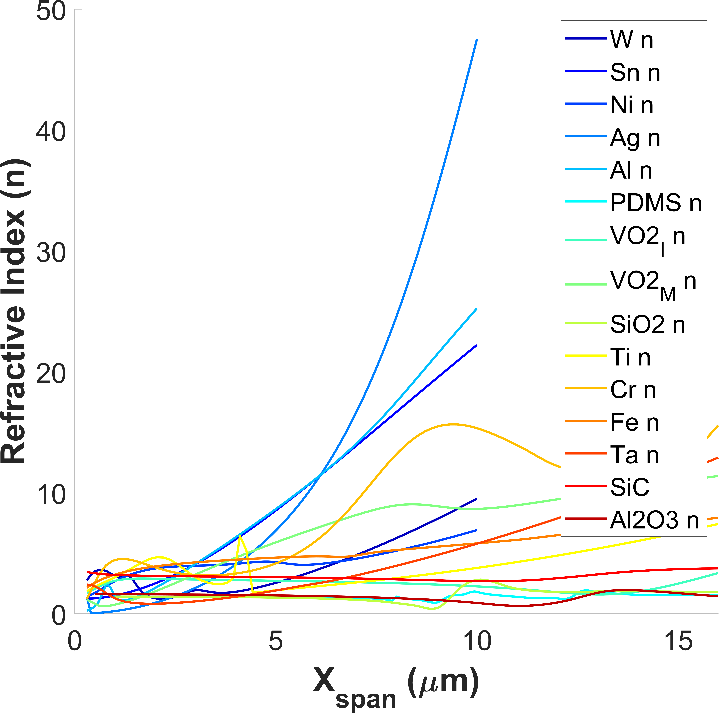
A wide array of materials are utilized in both the training of the neural network and the prediction of optical properties. How the model differentiates between materials is the same as an optical simulation, by discerning different values of the complex refractive index. We select a variety of materials for the training, ranging from metals (Ni/Ag/Al/Cr/Fe/Sn), refractory metals (Ta/W), a phase-change material (VO2 Metallic/Insulating), a polymer (PDMS), and a semiconductor (SiC). As shown in Figure S1, these materials have a wide range of complex refractive indices, making them excellent materials to use for training the model. Included in Figure S1 are titanium and alumina, materials that were not used in training but simulated for the unseen predictions. We plot

**
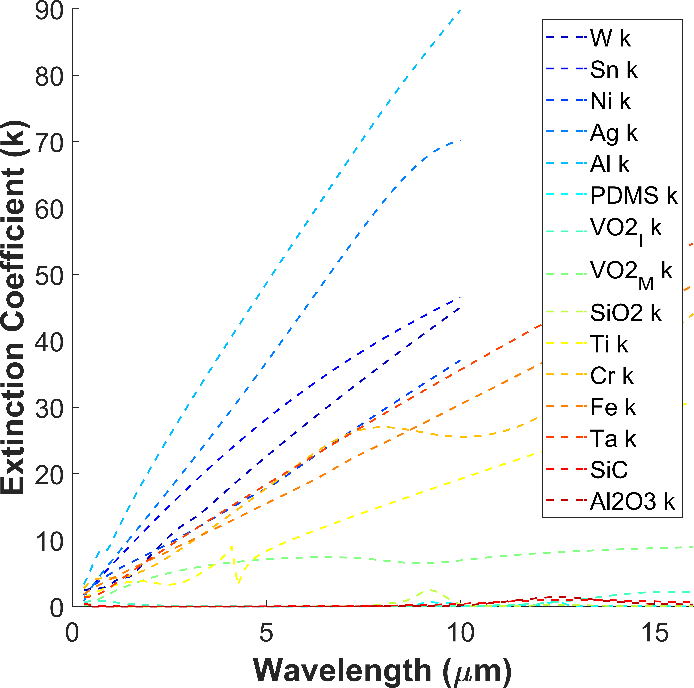
Figure S1.** The complex refractive index of materials used in training. (a) the refractive index and (b) extinction coefficient. Alumina (Al2O3) and titanium (Ti) are included for reference.

Titanium and alumina are plotted separately in Figure S2 compared to the rest of the materials to better show the comparison.


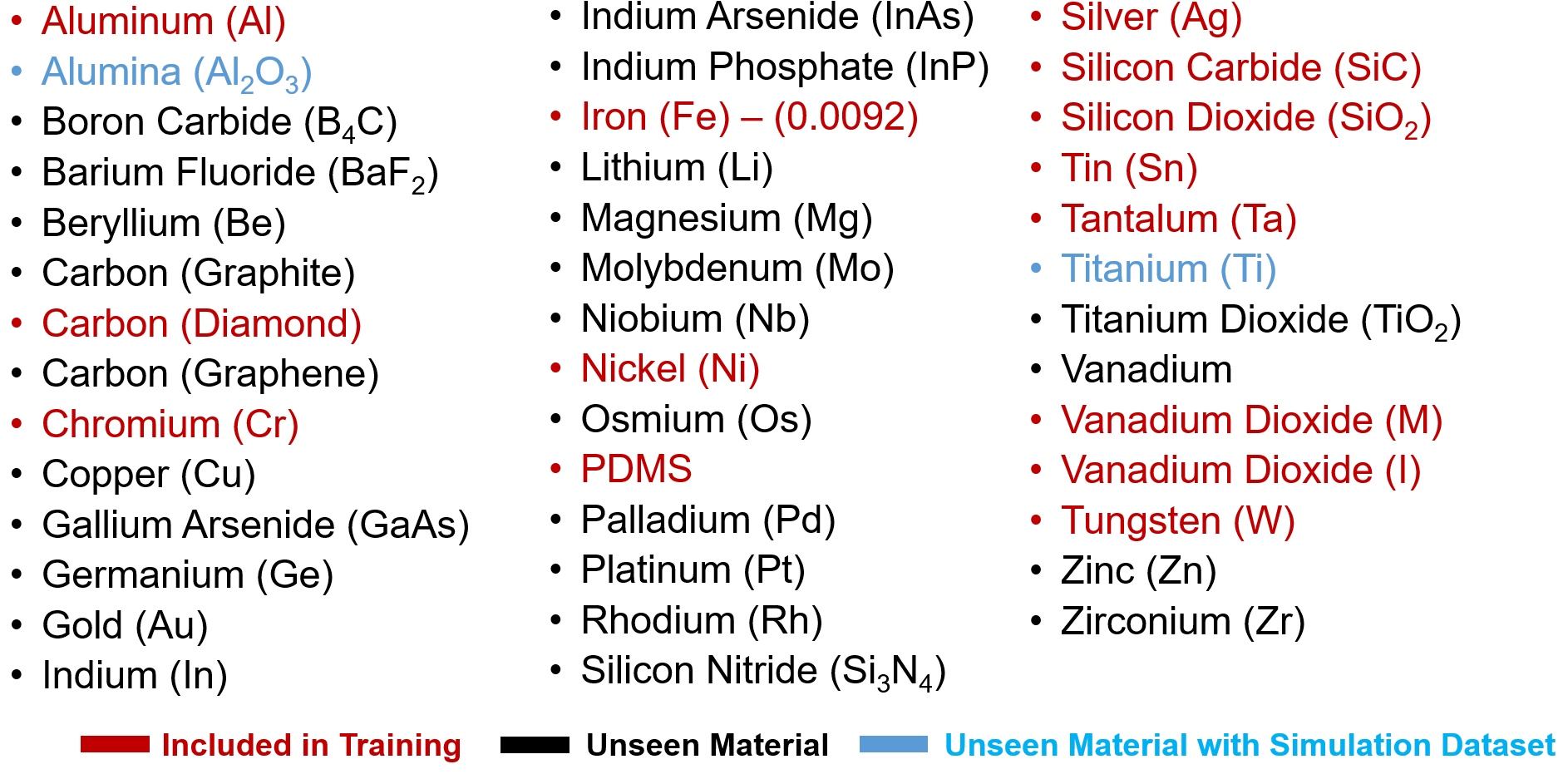


**Table S1.** List of all materials passed into the network for optical predictions. Included in the list are materials used in the training process and the two selected unseen prediction demonstration materials (Ti and Al2O3).

In total, we utilize a library of 41 materials for predictions. The comprehensive list of materials is shown in Table S1. In a similar fashion to the generation of the training datasets, we generate and simulate in FDTD 100 combinations of geometry for each of the unseen materials. We show the relative accuracy of these limited datasets in Figure 4 and Table 1 in the main text, both before and after “calibration” data is included in the training process. It should be noted that these materials were not chosen based on experimental validity or practicality of manufacture. Materials are chosen on a basis of difference from other materials and availability of material data over a wide spectrum of wavelengths. Not all material information extends from 0.3 to 16 um (the wavelength min/max used in training), so we simulate each material according to what is available for each. For the wide spectrum grid-geometry optical results used to generate the plots shown in Figure 5, we use the machine learning model to extrapolate the relationship between material and wavelength and provide predictions between 0.3 and 16 um.

**Figure S2.** Plot of the Refractive Index of Titanium and Alumina compared to the rest of the dataset. We plot the refractive index on the x-axis and extinction coefficient on the y-axis so as to better visualize the differences between the key neural network inputs from the training/test data. Both materials have distinct differences from the materials included in training, but do align with the physics of other metals and ceramics respectively.


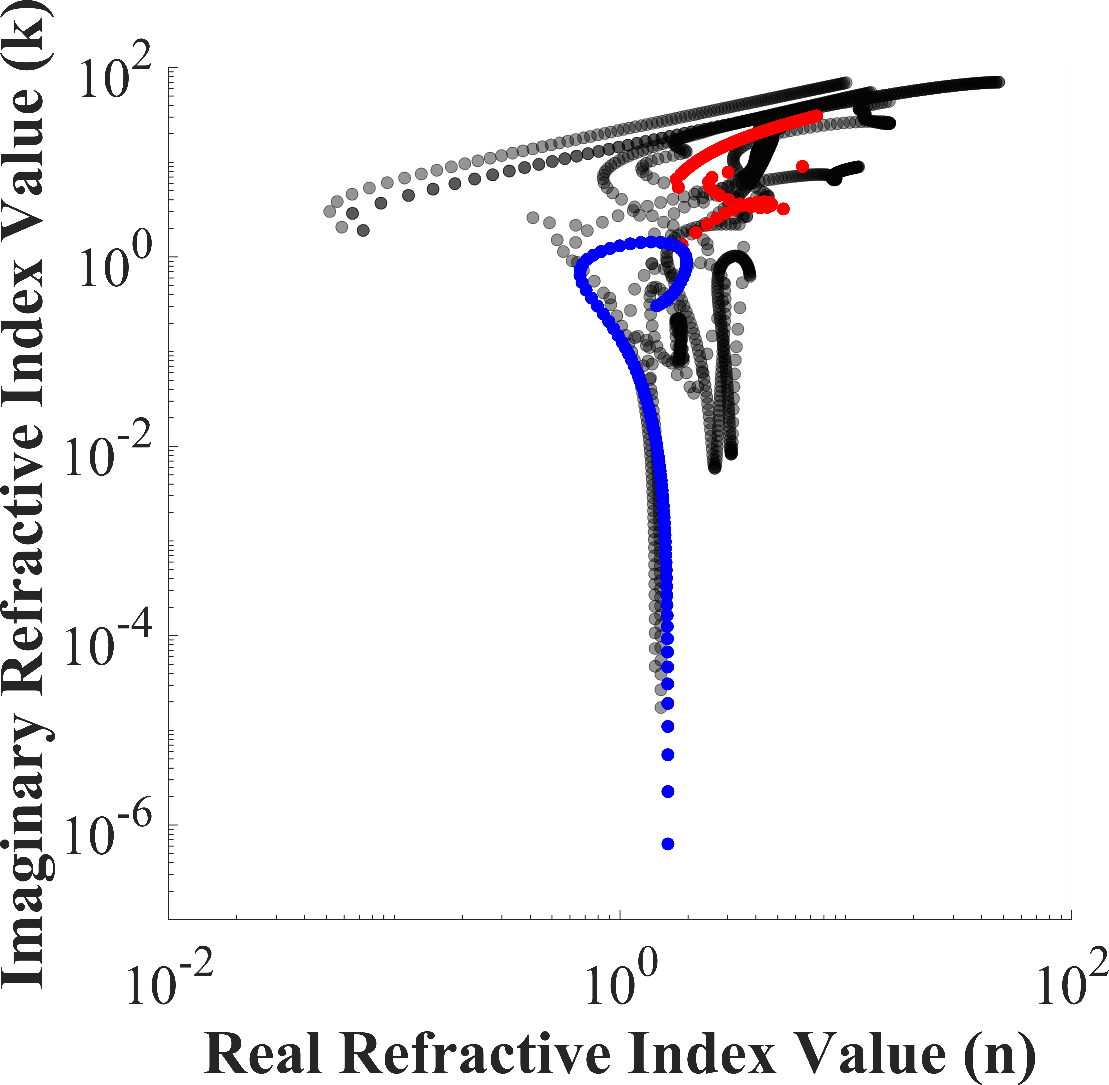


Training/Validation/Test Materials

Unseen - Titanium

Unseen – Al2O3

**Refractive Index (n)**

**Extinction Coefficient (k)**

**Section S2. Model Training, Validation, and Prediction Accuracy**

To ensure our model is not overfit, we track the validation and training MAE scores/loss over the course of the epochs run. Our models are capped at 200 epochs, but due to the selected checkpoints and callbacks we usually do not exceed 200 epochs in training. The values shown in Figure S3 are taken from the training of the model that has been used to generate the predictions shown in this work. Additionally, we show the predictions vs the ground truth for the test dataset shown in Figure 2 with more detail in Figure S4, demonstrating the accuracy of our approach for materials within the test/train/val dataset.

**b.)**

**a.)**


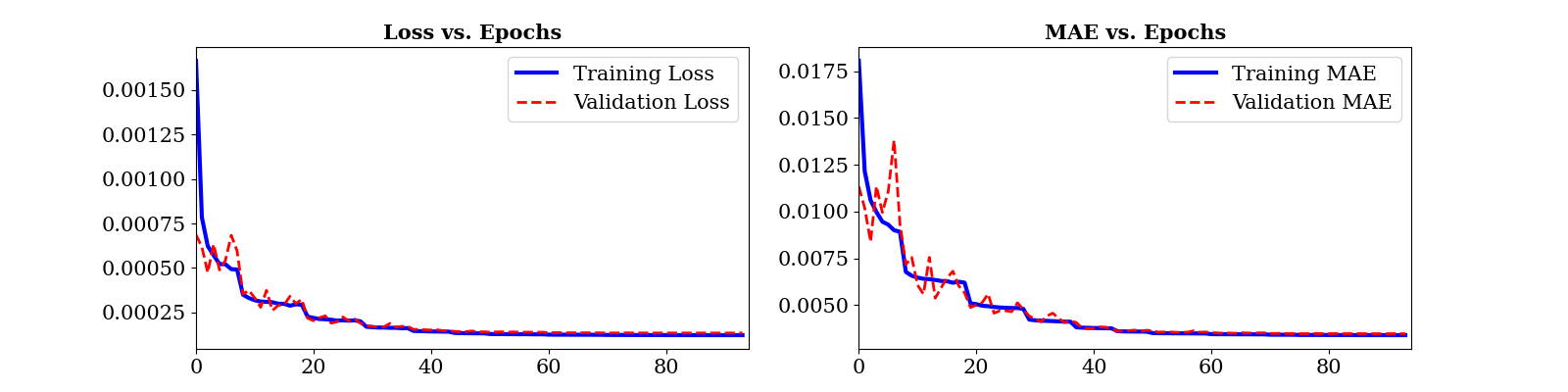

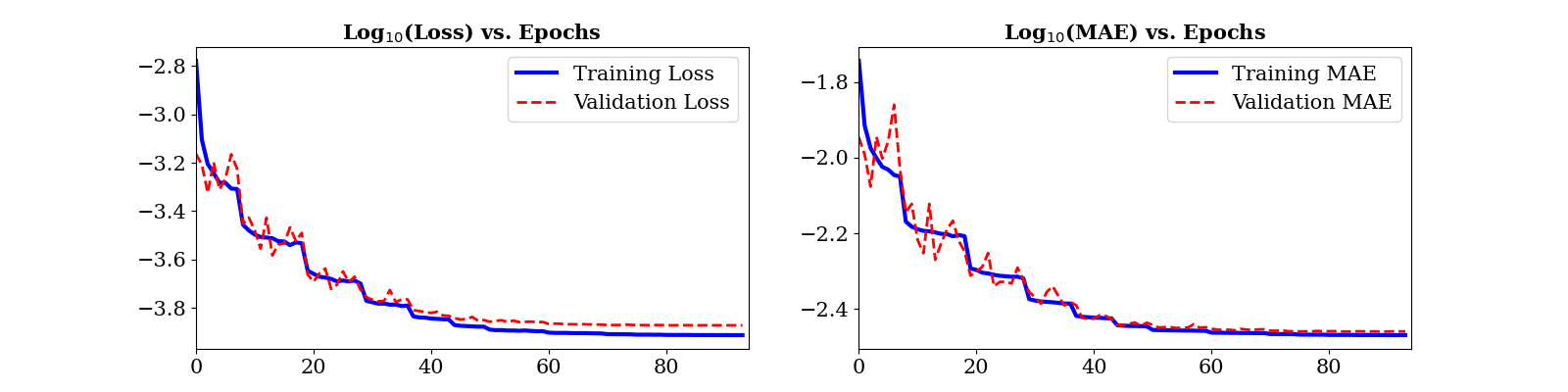


**d.)**

**c.)**

**Figure S3.** (a,b) Log10 Loss and MAE score shown across the training epochs. (c,d) MAE and Loss values shown over the course of the training epochs. It is observed that the training and validation loss/MAE scores do not deviate significantly. The MAE validation score of the best model is ~ 0.0032, which is only marginally lower than the test dataset (unused in training/validation) score of ~ 0.0034.

**
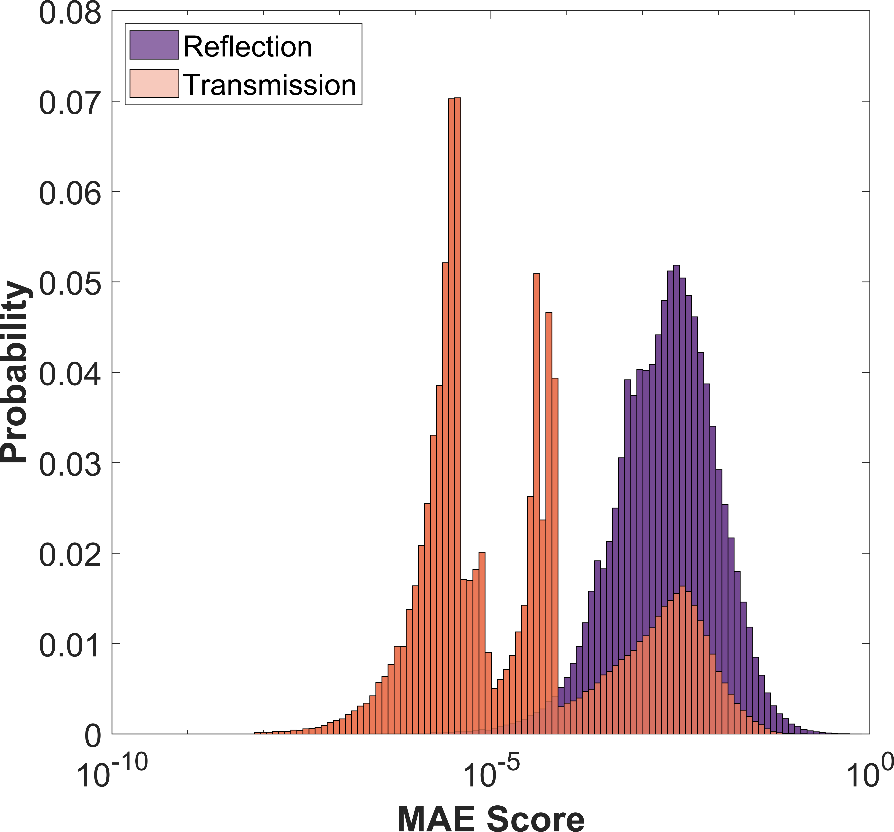
Figure S4.** Histogram distribution of MAE scores of the neural network predictions vs. the FDTD values for reflectivity and transmissivity in the test dataset with a logarithmic distribution. The distribution indicates that for most materials in the training dataset, the error is primarily attributed to differences in the reflection with a highly accurate predicted transmission.

**Section S3. Dataset Distribution and Normalization**


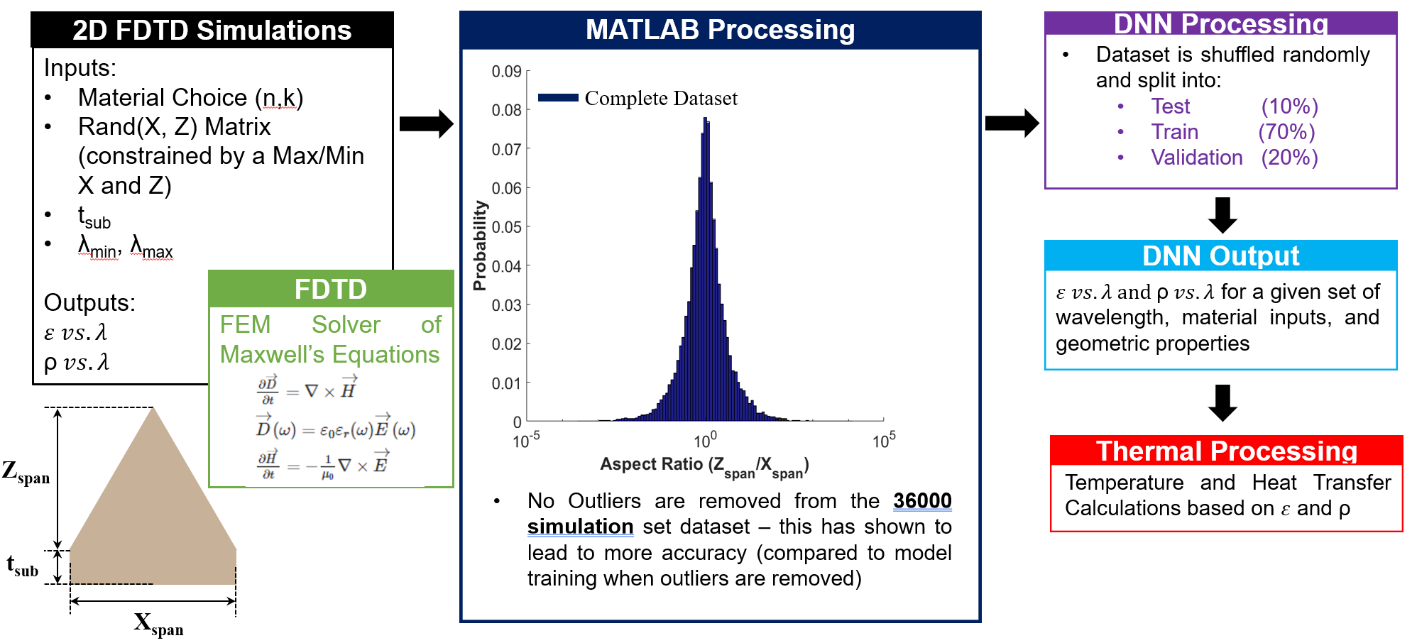
Figure S5 shows our process flow and distribution for generating the FDTD simulation data, normalizing the data into a dataset, splitting the dataset and using that dataset to train the model and then predict. The overall distribution of the random geometric matrices used to run the FDTD simulations is combined across all the materials and shown. The distribution for the geometric data is shown in terms of the aspect ratio (Z/X), the independent parameter that binds the X and Z span parameters of the micropyramids. While not used in the model, the aspect ratio is a good way to show geometric dependent optical properties. It is evident that the random process results in most of the pyramids having an aspect ratio near 1.

**Figure S5.** Process Flow Diagram of our simulation, dataset processing, DNN prediction, and post-processing phase. In total, for the 36000 simulations, 226 are eliminated for exceeding the aspect ratio cutoff, representing less than 1% of the simulations performed. This dataset is normalized using a linear normalization equation shown in the figure, and split into 3 components (Test, Train, and Validation) for the deep neural network. The Test dataset is used for later evaluation of the model (20%) and the training/validation datasets are used in the training process (80%).

Normalization is performed using multiple methods on an input-by-input basis, according to the original distribution to provide a more uniform dataset for each input parameter. The geometric input parameters for each FDTD simulation – xspan, and zspan – are generated using a uniformly distributed random process. The results are uniform random numbers, as shown in Figure S6. For these inputs, we use a simple linear scaling method to bring the geometric quantities between 0 and 1. All of the other dataset distributions are shown in Figure S7 before normalization. Due to the linearly spaced vector used for the wavelength, a simple linear normalization is effective for the wavelength input.

**
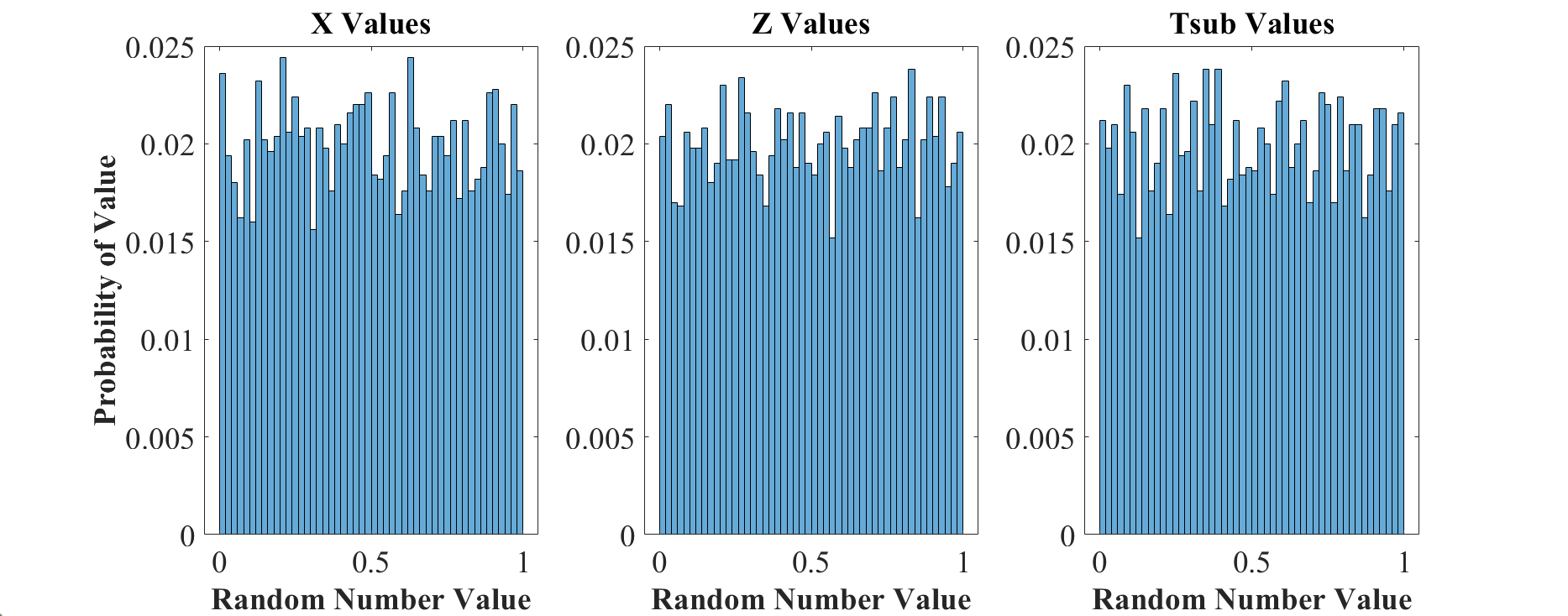
**
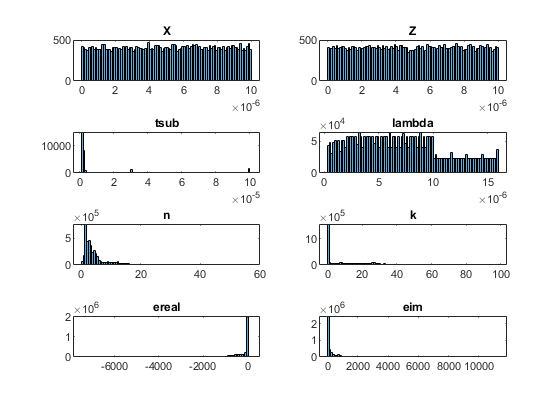
**Figure S6.** X and Z value distribution and validation of a random uniformly distributed geometric matrix. The min/max values of X/Z are set to 0 and 10 um respectively for all materials, while tsub varies by material (e.g, with metals ranging from 1 – 3 um, and ceramics from 1 – 100 um)

**Figure S7.** Distribution of the datasets before normalization. Although tsub is generated randomly, due to the large number of metals, the primary distribution is near 1 – 5 um.

While tsub is also randomly generated, its values depend on the constituent material and thus the thickness used in simulation is not random. For metals, we constrain tsub to be between 1 and 5 um, with other materials ranging from a maximum of 30 to 100 um depending on the transmission through the substrate. The values for n, k, ereal, and eim are all distributed near an extreme, due to many of the values being < 1 for the extinction coefficient (k). This provides a challenge for simple forms of normalization, and many methods were utilized and compared including Box-Cox and Yeo-Johnson methods. After many iterations, we found that a uniform quantile normalization led to the most accurate results when utilized for k, tsub, ereal, and eim. We utilize a simple log-normalization method for the n value, as quantile normalization for the n-value did not lead to a noticeable improvement. The normalized datasets are shown for n, k, tsub, ereal, and eim below in Figure S8.


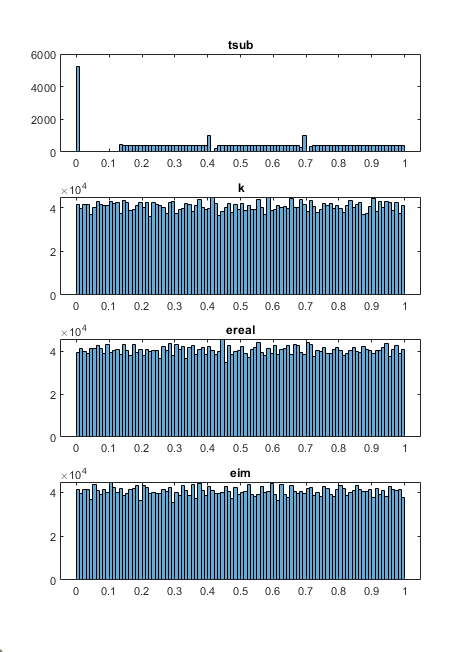

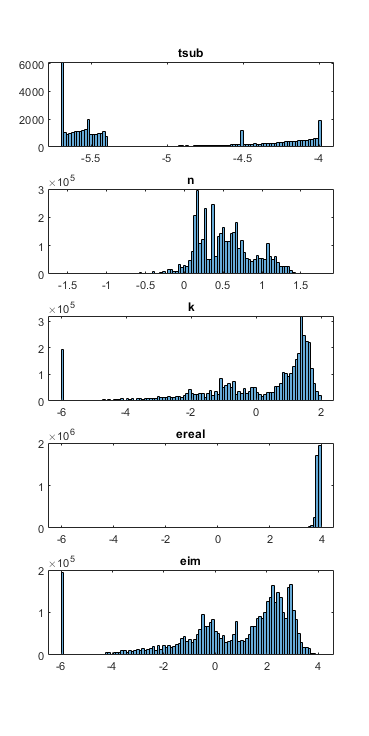

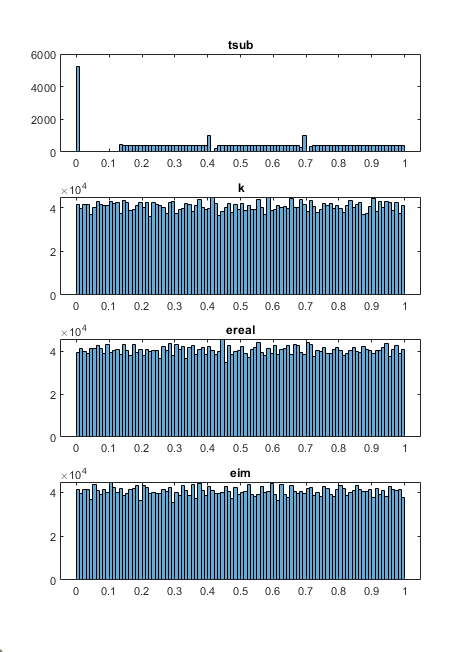

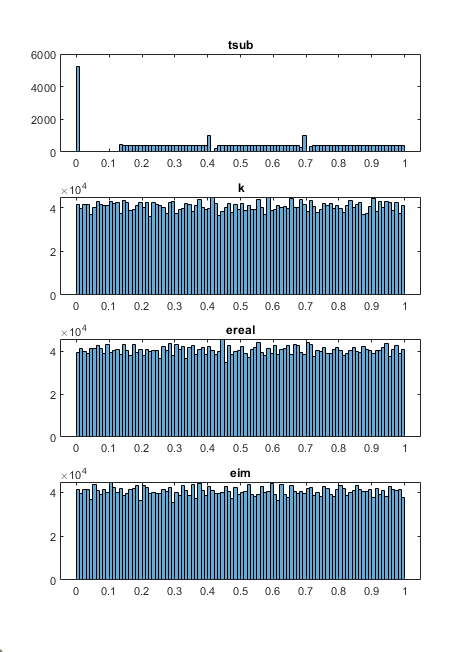

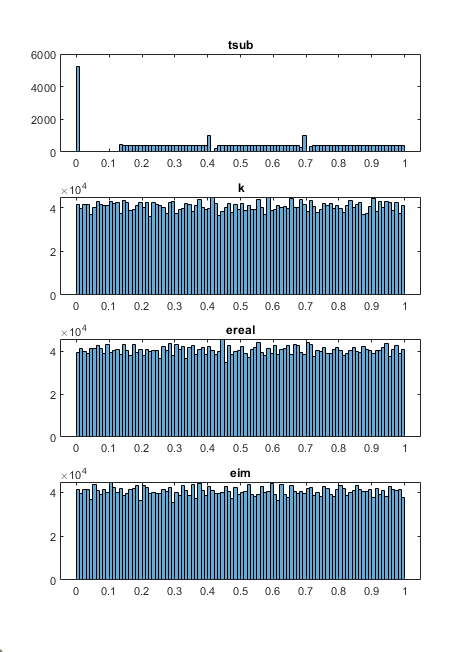


**Figure S8.** Datasets after normalization. tsub, k, ereal, and eim are all normalized using quantile normalization. To reduce complexity without sacrificing model accuracy, n is normalized using a simple log and then linear normalization method.

**Section S4. Design Space for Each Material**

Despite having only three independent geometric variables (X_span_, Z_span_, t_sub_) the design space for each material simulated is extensive. As shown in Figure S9, which is generated from the test dataset, while the metallic materials have a strong dependence upon the aspect ratio, the polymer/ceramic material have both a dependence on the aspect ratio as well as a strong dependence on the thickness of the substrate. The design space for this microstructure is visualized in another form in Figure 9(b) and Figure 9(c), by giving a 3D surface visualization of the calculated emissivity (E = 1 – R – T) from the neural network. The strong diversity in solutions across the geometric parameters we have identified, when combined with material properties and wavelength, lead to a large design space for thermal applications and highlights the importance of having a rapid design tool.


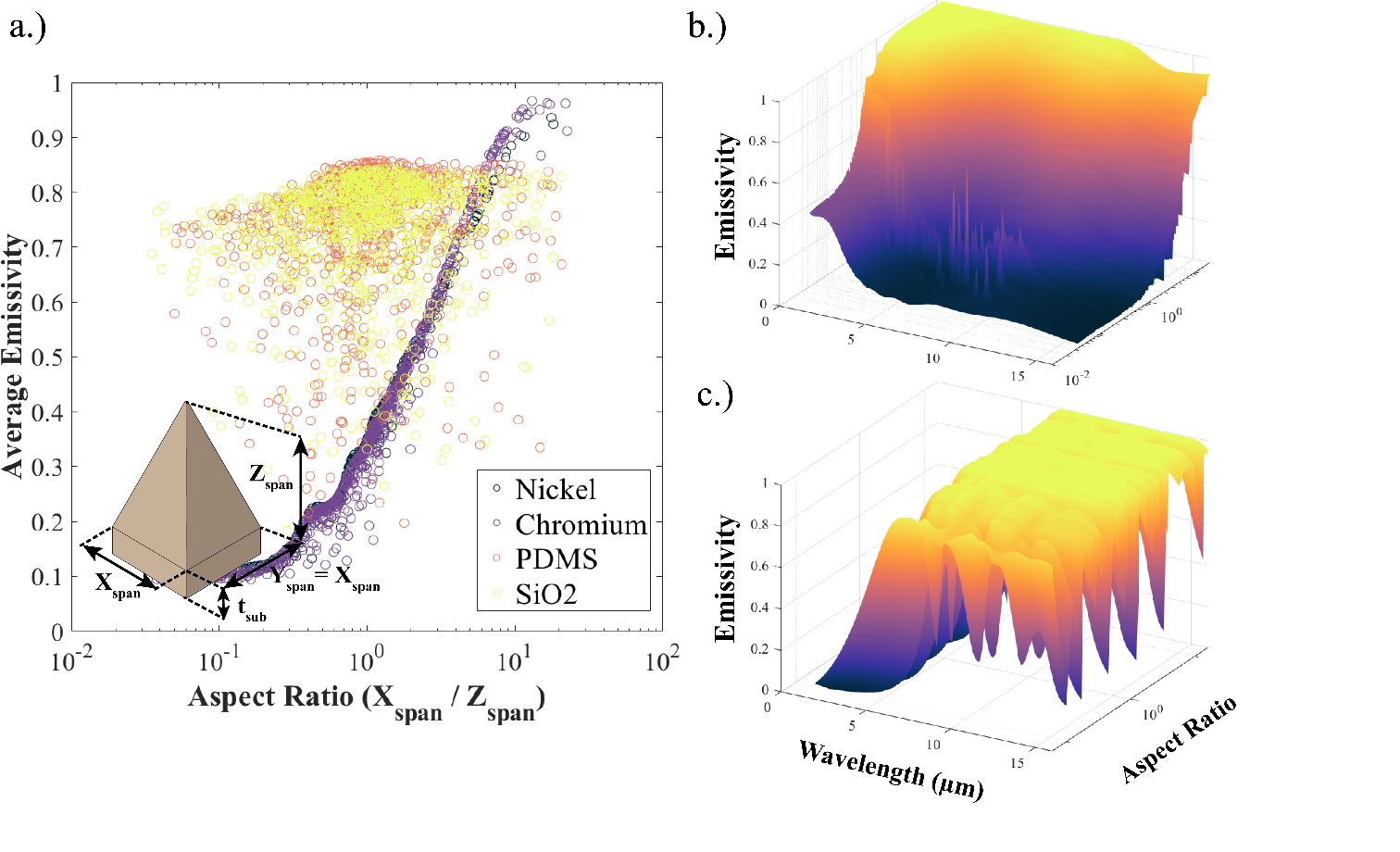
**Figure S9(a)** Visualization of design space for four materials – two metals (Ni/Cr), a polymer (PDMS), and ceramic (SiO2) – with average emissivity plotted with respect to the pyramid aspect ratio. Metals demonstrate a consistent increase in average emissivity as the design aspect ratio increases whereas the polymer and ceramic materials have a stronger dependence on both substrate thickness and x_span_. **(b)** Surface plot of emissivity of Cr with the wavelength on the x-axis and aspect ratio on the y-axis. Low aspect ratio peaks result due to localized resonance. **(c)** Emissivity of PDMS plotted with wavelength on the x-axis and aspect ratio on the y-axis.

**Section S5. Thermal Predictions – FDTD and Simulation**

Similar to Figure 3, the emissivity data can be translated into thermal predictions using simple (or complicated) thermal optimization equations. The result of this process enables us to identify optimum geometries that best suit the thermal optimization equations and quick evaluation and prediction of thermal performance for an engineering microstructure. The error shown between the optical predictions made by the network vs the simulated values is shown in Figure 3, but we mimic the same procedure for the thermal processing in Figure S10. We have 1500 simulations for both titanium and alumina, so much like Figure 3 we take the calculated emissivity from the simulations and the calculated emissivity from the network to make thermal predictions and compare the error in the figure of merit. The small difference between the two indicates that while the optical value error may be large at some points, this does not translate necessarily to large error in the thermal predictions. For both datasets, the error in the computed FOM does not exceed 0.15.


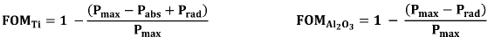

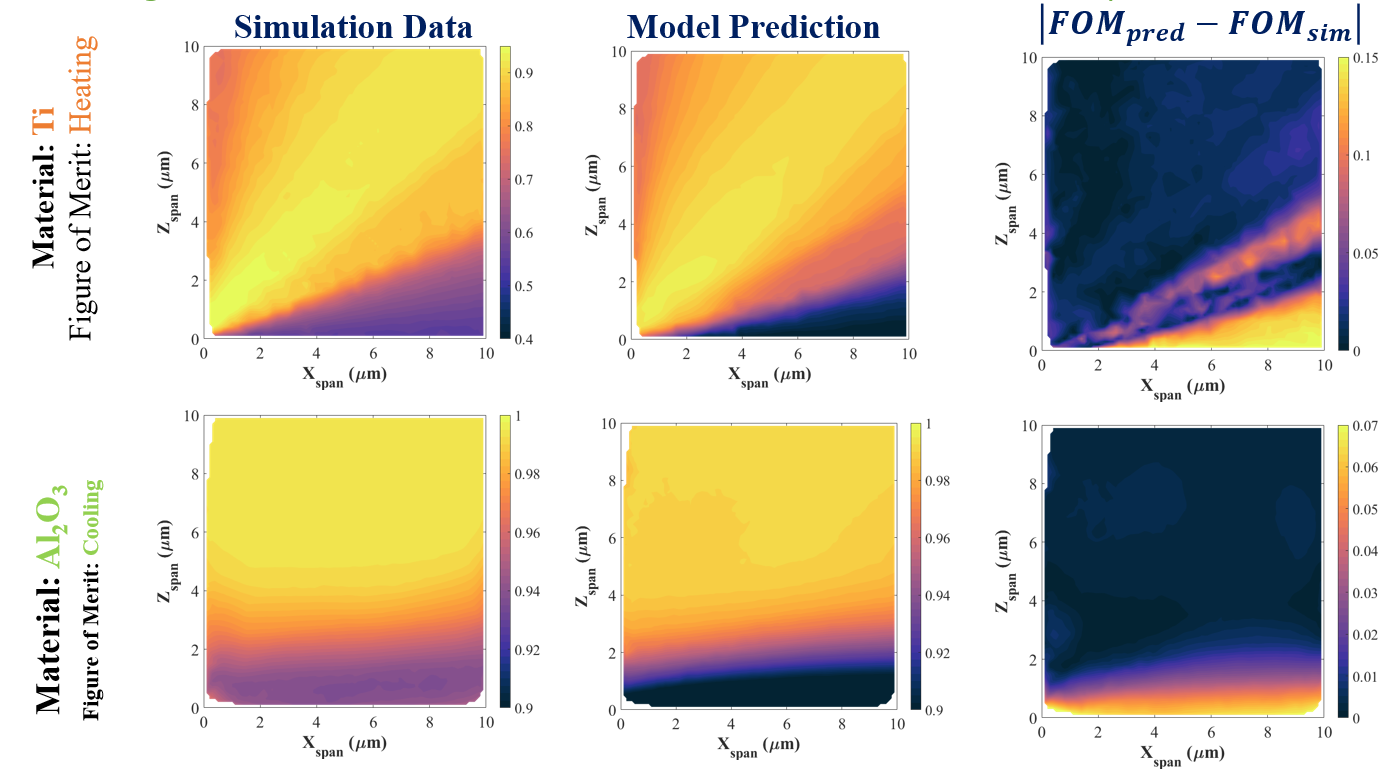


**Figure S10.** The Figure of Merit computed using the emissivity data generated by simulations, predictions, and then the relative error between the two. The maximum error in the titanium dataset is 0.15, but this error is mostly confined to lower aspect ratio structures in the bottom right corner. This behavior is also observed in alumina. For most of the predictions, there is a negligible difference between the thermal computations performed using emissivity data from the neural network vs the thermal computations performed via the FDTD simulation data.

**Section S6. Material Search Algorithm – Identifying Geometries for Fabrication and Thermal Optimization**


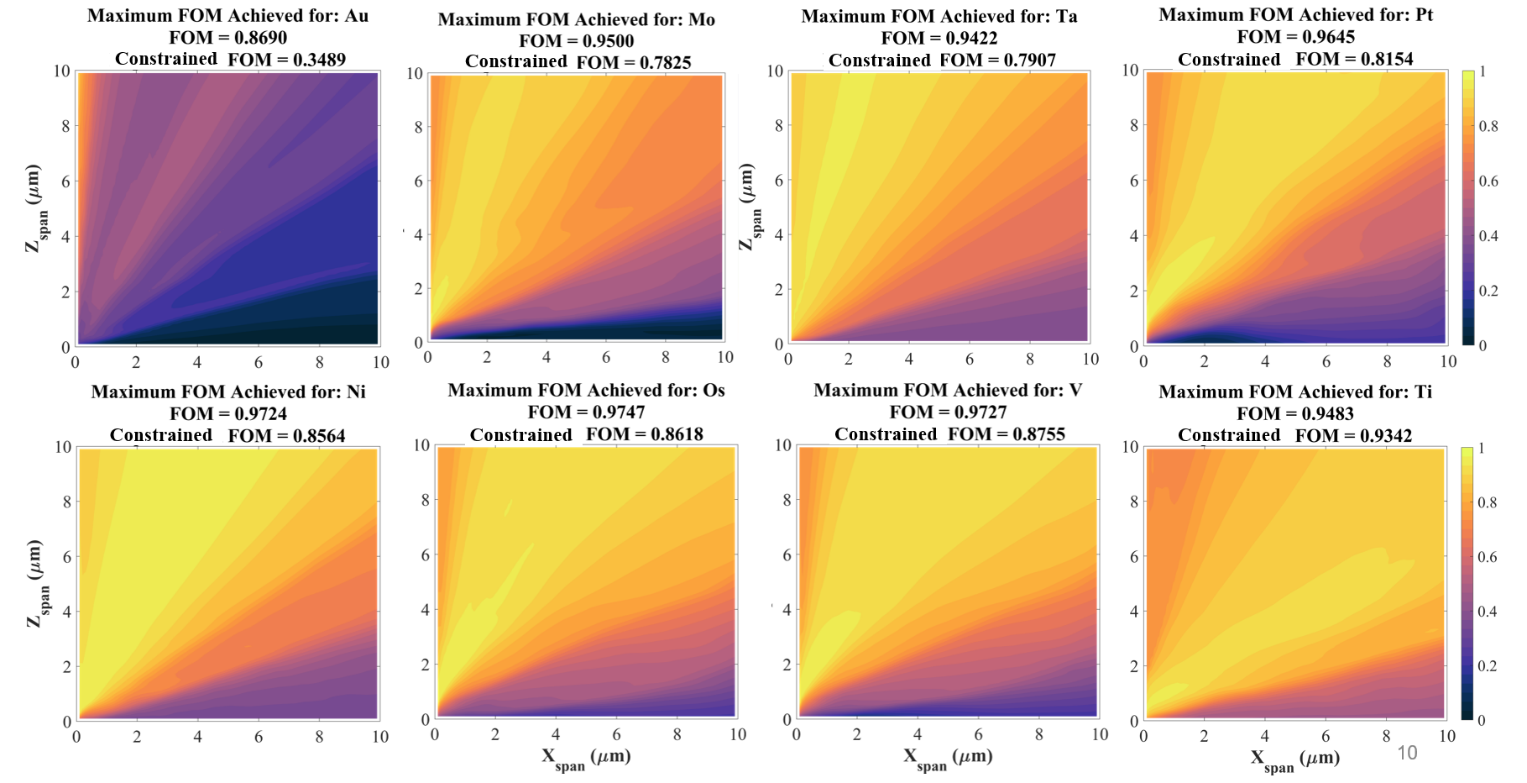
The ability to generate hundreds of thousands of simulations synthetically in seconds means we can perform comprehensive optimizations that would not be possible previously. We can set constraints on the data generated and then subsequently search the entire set of generated optical properties within the confines of the set constraints. An example of previously unrealistic optimization that is now possible via our neural network is in the identification of optimal geometries that fall under a particular aspect ratio. The scalable methodology used to fabricate the base silicon molds has a process limited aspect ratio of ~ 0.8, so being able to identify not only what geometry but also what material leads to the best thermal result across all the materials simulated and within that constraint would not have been possible previously. In Figure S11, we demonstrate the identification of the best constrained geometries based on the heating figure of merit shown in Figure S10.

**Figure S11**. Thermal prediction plots generated using the heating figure of merit shown in Figure 8 for titanium and discussed in the methods section. The predictions are done using the 10,000 synthetic simulation reflectivity/transmissivity predictions done by the neural network. This process identifies titanium has having the best radiative heating performance across all materials when the aspect ratio of the structure is constrained to < 0.85, with a value of 0.934. This is compared to Os/Ni which are identified as two of the best heating materials when aspect ratio is not constrained, at 0.975/0.972 respectively. When their aspect ratios constrained to a value less than 0.85, they demonstrate a substantially lower maximum figure of merit.

This process enables us to provide thermal optimizations for a wide array of solutions to complex thermal problems. An often-studied application of micropyramids is for high-temperature heating or radiative heating applications. Nickel – a highly mechanically and optically versatile metal – is a key area of research foucs. Using the neural network, we can generate optical solutions for many metals and compare them to one another for a radiative heating application, in particular to see how nickel performs compared to other metals. We find that nickel – among the materials that we have selected to study – is a top-performing metal, suggesting that it is likely one of the best suited metals for radiative heating applications.

**
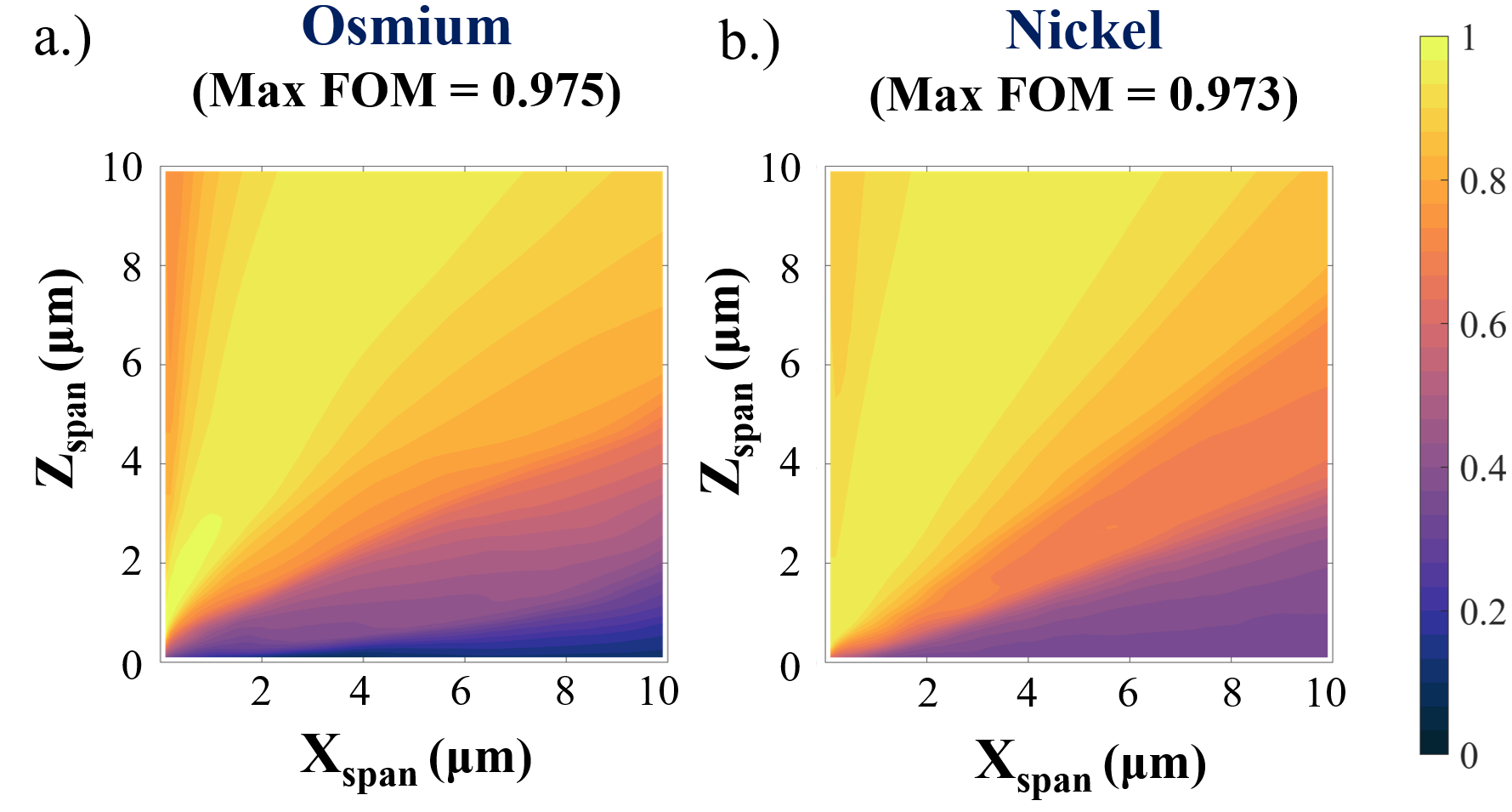
Figure S12** Identifying the micropyramid material that best optimizes the heating figure of merit (Eq. 4) at a surface temperature of 300 K. The predictions made by the neural network for the geometric grid (10,000 simulations per material) were used as the basis of the wavelength dependent emissivity in Eq. 5. (a) Our network emulated optical data projects that Osmium micropyramids would be the most optimal material for radiative heating. (b) Nickel, a common engineering material that is often used for micropyramids in previous work, is projected to perform nearly as well as Osmium.

**Section S7. Model Iteration Process**

Our modelling efforts underwent extensive modification to arrive at the current accuracy. Initially, as described in Table S2, our network was built upon the full simulation wavelength spectrum. For this setup, while we only had 7 classifications of inputs, we had 304 input neurons – 100 wavelength points, and 200 corresponding n and k values, one for each wavelength. In this original setup, we also utilized the aspect ratio as an additional geometric parameter. Future iterations determined that we could significantly improve our accuracy by changing to a singular input set (i.e, one wavelength/n/k value), and further improve the connection between the input and output via additional material parameters (ereal, eim). Large improvements to the accuracy were also made by paying particular attention to the normalization method, and many methods were utilized and compared. Our end result is described in a previous section but is a mixture of normalization methods for each input. Finally, to further improve the connection between the input and output, we separated the geometric and material inputs and first run them through two separate MLPs that ultimately connect to the larger DNN. All these steps allow the model to greatly increase its understanding the relevant physics and make more accurate predictions for a new and unseen set of material information, wavelength, and geometry.

| **Gen** | **Inputs*** | **Input**  **Neurons** | **Architecture** | | **Normalization** | | **Outputs** | | **Type of Output**** | **Library Accuracy** |
| --- | --- | --- | --- | --- | --- | --- | --- | --- | --- | --- |
| 1 | 7 | 304 | | DNN | | Linear | | 200 | E, R | 0.105 |
| 2 | 7 | 304 | | DNN | | **Linear/Log** | | **200** | **R, T** | 0.095 |
| 3 | **6** | **6** | | DNN | | Linear/Log | | 2 | R, T | 0.068 |
| 4 | **8** | **8** | | DNN | | Linear/Log | | 2 | R, T | 0.049 |
| 5 | 8 | 8 | | DNN | | **Lin/Log/Quant** | | 2 | R, T | 0.032 |
| 6 | 8 | 8 | | **2 MLP + DNN** | | Lin/Log/Quant | | 2 | R, T | 0.027 |

* Number of Inputs: 7 - (X, Z, AR, tsub, lambda, n, k)

6 - (X, Z, tsub, lambda, n, k)

8 - (X, Z, tsub, lambda, n, k, ereal, eim)

9 - 2 sets of inputs: (X, Z, tsub, lambda) and (lambda, n, k, ereal, eim)

** Output Type: Emissivity (E), Reflectivity (R), and Transmissivity (T)

**Table S2.** Comparison of successive model iterations, comparing the differences in model architecture, output type, input type, and ultimately the model’s accuracy in predicting the library of materials. The final generation – the one shown in the paper – performs exceptionally well, predicting the optical properties of micropyramids constructed from materials not included in training with a high degree of accuracy. Changes from generation to generation are bolded.

**Section S8. Minimal Overfitting Validation**

To show that our test dataset accuracy is neither overfitting nor predicting off of already seen data, we compare overlap between the test dataset and training dataset. The entire simulation dataset has 35500 simulations: the training data contains 24850 simulations (70%), the validation dataset contains 7150 simulations (20%), and the test dataset contains 3550 simulations (10%). Before we input the data into the model – whether we are training it or not – the datasets are randomized, so no train/test/validation dataset should ever be identical. To quantify dataset overlap, we randomize/split our simulations 10,000 times to find the mean overlap between the test and training/validation datasets. The results for the overlap in matching X/Z pairs between the test and training/validation datasets are shown below in Figure S13. Over the 10,000 shuffled iterations, we found a mean value of 287 overlaps of X/Z pairs between the test and training/validation datasets. That is, approximately < 10% on average of the test dataset shares a pair with the training/validation dataset. When we extend the search for matching X,Z, and tsub triples, however, we find that there is no overlap between the test and training/validation datasets. Thus, we can conclude with high confidence that the test data is indeed distinctly different from the training/validation data.

**
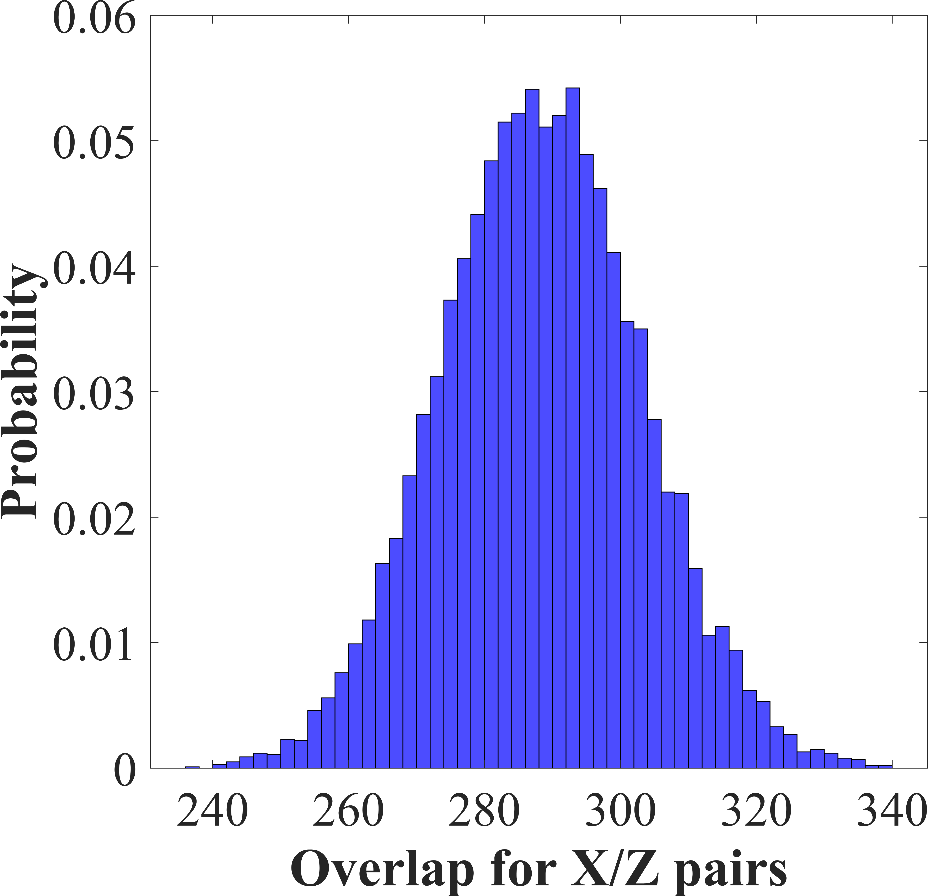
Figure S13.** Histogram of the computed overlap of X/Z between the test and training/validation datasets over 10,000 different shuffles. The integer mean value of overlap was 287, less than 10% of the dataset. When we look for overlap between triples of the geometric information, we find no overlap between test and training/validation datasets.
